# The Cipher Code of Simple Sequence Repeats in “Vampire Pathogens”

**DOI:** 10.1038/srep12441

**Published:** 2015-07-28

**Authors:** Geng Zou, Bernardo Bello-Orti, Virginia Aragon, Alexander W. Tucker, Rui Luo, Pinxing Ren, Dingren Bi, Rui Zhou, Hui Jin

**Affiliations:** 1 State Key Laboratory of Agricultural Microbiology, College of Veterinary Medicine, Huazhong Agricultural University, Wuhan 430070, China; 2 Centre de Recerca en Sanitat Animal (CReSA), UAB-IRTA, Campus de la Universitat Autònoma de Barcelona, 08193 Bellaterra, Spain; 3 Institut de Recercai Tecnologia Agroalimentàries (IRTA), Barcelona, Spain; 4 Department of Veterinary Medicine, University of Cambridge, Cambridge, United Kingdom

## Abstract

Blood inside mammals is a forbidden area for the majority of prokaryotic microbes; however, red blood cells tropism microbes, like “vampire pathogens” (VP), succeed in matching scarce nutrients and surviving strong immunity reactions. Here, we found VP of *Mycoplasma*, *Rhizobiales*, and *Rickettsiales* showed significantly higher counts of (AG)_n_ dimeric simple sequence repeats (Di-SSRs) in the genomes, coding and non-coding regions than non Vampire Pathogens (N_VP). Regression analysis indicated a significant correlation between GC content and the span of (AG)_n_-Di-SSR variation. Gene Ontology (GO) terms with abundance of (AG)_3_-Di-SSRs shared by the VP strains were associated with purine nucleotide metabolism (*FDR < *0.01), indicating an adaptation to the limited availability of purine and nucleotide precursors in blood. Di-amino acids coded by (AG)_n_-Di-SSRs included all three six-fold code amino acids (Arg, Leu and Ser) and significantly higher counts of Di-amino acids coded by (AG)_3_, (GA)_3_, and (TC)_3_ in VP than N_VP. Furthermore, significant differences (*P < *0.001) on the numbers of triplexes formed from (AG)_n_-Di-SSRs between VP and N_VP in *Mycoplasma* suggested the potential role of (AG)_n_-Di-SSRs in gene regulation.

Successful proliferation of prokaryotic pathogens parasitizing in the red cells of blood [hereinafter as VP (vampire pathogens) versus N_VP (non vampire pathogens)] implies that VP have developed strategies to match a multitude of dangerous host defense mechanisms, such as complement-mediated cytolysis, phagocytosis and antimicrobial peptide-mediated killing. Correspondingly, the spectrum of VP resistance mechanisms includes antigenic variation, capsule biosynthesis and modification of host cell surface. Notably, hemotrophic prokaryotic pathogens need to adjust their metabolism to the scarce nutrients in blood, including limiting concentrations of purine, nucleotide bases and iron during the course of infection^1^. Despite advances in sequencing and analyzing the genomes of VP, little is found about the determinants in gene content through the selective evolution to survive in the blood.

Being deemed the product of organic evolution, simple sequence repeats (SSRs) composed of tandem iterations of short oligonucleotide are subject to slipped-strand mutations in prokaryotes^2^,^3^. Phase variation is a microbial survival strategy that relies on stochastic, reversible on–off switching of gene expression with SSRs to facilitate avoidance of host immune response^3^,^4^. Also, long SSRs in *Mycobacterium leprae* mostly associated with pseudogenes may contribute to gene loss for the adaptation to an obligate pathogenic lifestyle^5^. Previous study found few statistically significant associations between SSRs in obligate pathogens and gene functional classifications, resulting in researchers got confused about the bond between long SSRs with a particular gene function^5^. However, in this study, a plenty of significant GO terms (*FDR* < 0.01) were identified associated with VP (AG)_3_-Di-SSRs.

*Mycoplasma* is an optimum model for studies of gene function because it possesses a minimalistic and economical genome for basic biosynthetic capabilities and parasitic lifestyle^6^. Subtle changes in the *Mycoplasma* genome can reflect essential requirements in the face of survival pressure. Here we began with the analysis of Di-SSR in *Mycoplasma*, and then further explored the research scope to VP of the other prokaryotic microbe orders and mechanism by which VP developed specific Di-SSR during the course of evolution.

## Results and Discussion

### (AG)_n_-Di-SSRs abundance in *Mycoplasma* VP

**Figure 1:**
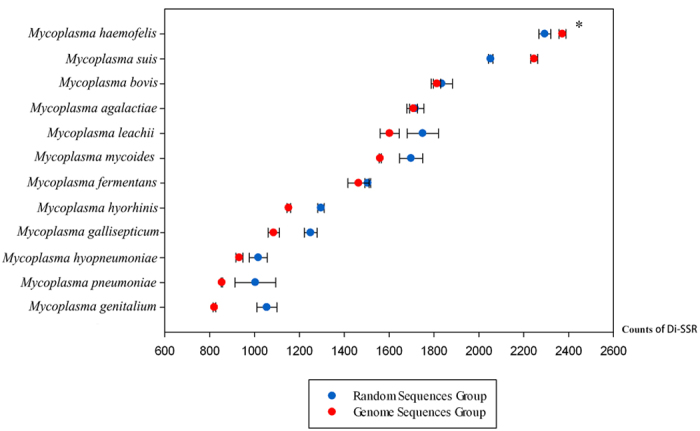
The evolution of Di-SSRs in *Mycoplasma*. ^*^The numbers of Di-SSRs in artificial random *Mycoplasma* genomes of 12 species showed higher standard deviations in comparison to the real genomes at *P* < 0.05.

**Figure 2:**
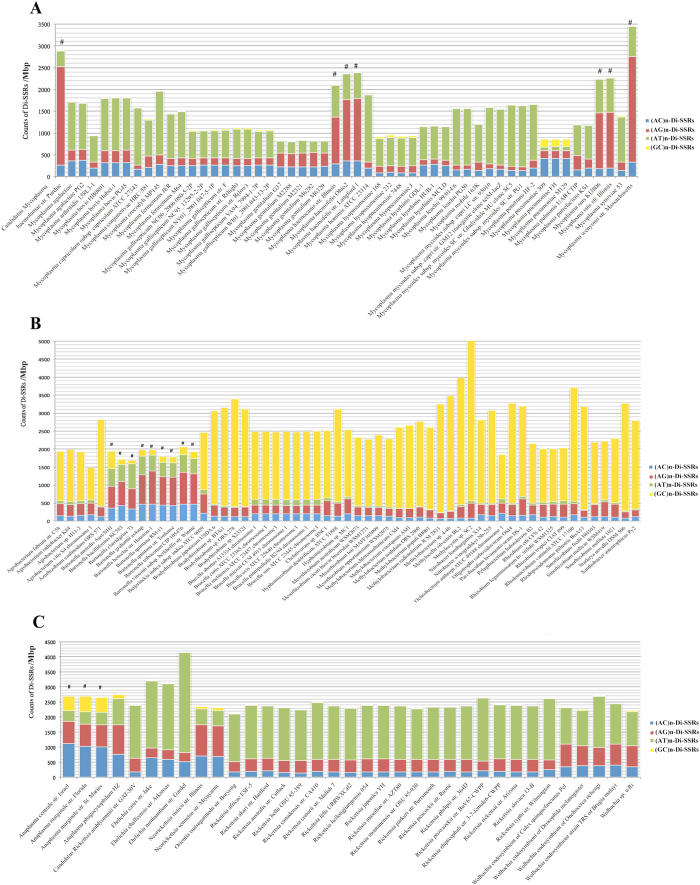
Density distribution of Di-SSRs in the genomes of *Mycoplasma*, *Rhizobiales* and *Rhickettsiales*. **A** *Mycoplasma*, **B** *Rhizobiales* and **C** *Rickettsiales*. ^#^Vampire Pathogens (VP).

A pairwise comparison was performed between twelve real *Mycoplasma* genomes and their corresponding artificial random genomes. The heterogeneity of the real genome sequences was eliminated in the artificial random DNA genome sequences, which mimicked the genomes uninfluenced by evolutionary pressure. This comparison showed the variable distances of Di-SSRs in the actual genomes were significantly wider than the estimated divergence (*P* < 0.05) (Fig. 1), suggesting Di-SSRs variability in *Mycoplasma* is the product of selective evolution, not a stochastic process. Furthermore, we used a length cutoff of 6 bp, as previous studies of SSRs in prokaryote^7^ and analyzed the density distribution of four subtypes of dimeric SSRs in 53 fully sequenced *Mycoplasma* strains. Interestingly, *Mycoplasma* strains isolated from the blood of different mammalian species (Supplementary Table S1) showed significantly higher counts of (AG)_n_-Di-SSRs in comparison to others in genomes, coding regions and non-coding regions (Fig. 2A and Supplementary Table S2), which brought up the hypothesis that (AG)_n_-Di-SSRs accumulated to benefit VP survival in blood. Towards eliminating the influence of difference in genome length, the count of Di-SSRs was normalized to the size of the genome, coding regions and non-coding regions. Di-SSRs of (AG)_3_, (AG)_4_, and (AG)_5_ were observed in all seven VP genomes, and (AG)_3_ repeats presented an overwhelming majority of the repeat units (Supplementary Table S3).

### (AG)_n_-Di-SSRs abundance in *Rhizobiales*, and *Rickettsiales* VP

To examine if our hypothesis applies only to VP in *Mycoplasma* or is more generalizable, we evaluated the fully sequenced genomes of all nine VP *Bartonellaceae* strains to the other 48 *Rhizobiales* strains and 3 VP *Anaplasma* strains to the other 32 Rickettsiales strains. Notably, the results again showed significantly higher counts of (AG)_n_-Di-SSRs in the genomes, coding regions and non-coding regions, which confirmed the importance of (AG)_n_-Di-SSRs in blood parasites (Fig. 2B, 2C and Supplementary Table S2).

### The relationship between GC content and Di-SSRs

**Figure 3:**
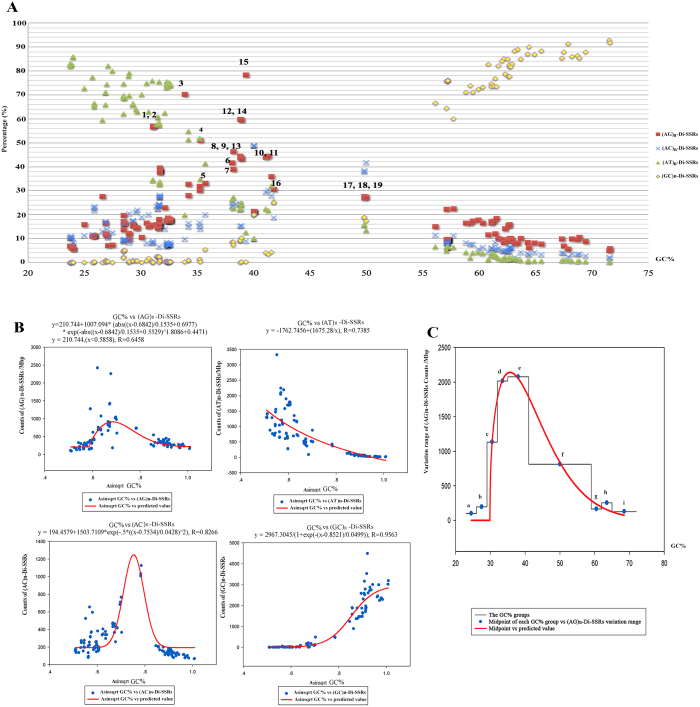
Analyses of relationship between GC content and Di-SSRs in the genomes of *Mycoplasma*, *Rhizobiales* and *Rhickettsiales*. **A** Percentages of each Di-SSRs subtype in relation to GC % in the genome of *Mycoplasma*, *Rhizobiales* and *Rickettsiales*. ^1–19^Vampire Pathogens (VP). ^1^*Mycoplasma suis* KI3806, ^2^*Mycoplasma suis* str. Illinois, ^3^*Mycoplasma wenyonii* str. Massachusetts, ^4^*Mycoplasma haemocanis* str. Illinois, ^5^*Bartonella* clarridgeiae 73, ^6^*Bartonella grahamii* as4aup, ^7^*Bartonella bacilliformis* KC583, ^8^*Bartonella henselae* str. Houston-1, ^9^*Bartonella quintana* RM-11, ^10^*Bartonella quintana* str. Toulouse, ^11^*Bartonella vinsonii* subsp. berkhoffii str. Winnie, ^12^*Mycoplasma haemofelis* Ohio2, ^13^*Bartonella tribocorum* CIP 105476, ^14^*Mycoplasma haemofelis* str. Langford 1, ^15^*Candidatus Mycoplasma haemolamae* str. Purdue, ^16^*Bartonella australis* Aust/NH1, ^17^*Anaplasma marginale* str. Florida, ^18^*Anaplasma marginale* str. St. Maries, ^19^*Anaplasma centrale* str. Israel. **B** The correlation between the counts of each subtype of Di-SSRs and the GC % was analyzed by nonlinear regression. **C** Analysis of the correlation between GC % and variation range of (AG)_n_-Di-SSRs. Intervals of GC %: a, 23.0 to 26.0%. b, 26.0 to 29.0%, c, 29.0 to 32.0%. d, 32.0 to 35.0%. e, 35.0 to 41.0%. f, 41.0 to 59.0%. g, 59.0 to 62.0%. h, 62.0 to 65.0%. i, 65.0 to 72.0%.

Considering the above observations, we further explored the (AG)_n_-Di-SSRs presentation of individual genome in VP in comparison to the N_VP in the same taxonomic class by using Chebyshev’s inequality. The results showed that the count of (AG)_n_-Di-SSRs in VP significantly deviated from the norm with exception of three *Anaplasma* strains (Supplementary Table S4), which had a remarkable higher GC content relative to the other VP (Supplementary Table S1). A series of analyses were developed to understand the relationship between GC content and Di-SSRs. Figure 3A showed the percentage of each subtype of Di-SSRs per genome in relation to the GC content of 145 strains of *Mycoplasma*, *Rhizobiales* and *Rickettsiales*. VP with the GC content between 31.1% to 41.8% showed high levels of (AG)_n_-Di-SSRs and moderate levels of (AC)_n_-Di-SSRs, while the three *Anaplasma* strains (about50% GC) showed moderate levels of (AG)_n_-Di-SSRs and high levers of (AG)_n_-Di-SSRs (Fig. 3A). Regression analysis revealed that the relative content of (GC)_n_-Di-SSRs was strongly influenced by genome GC content. However, the lower R value of the regression equation of (AG)_n_ and (AC)_n_Di-SSRs indicated that the GC content was not the only determinant of SSR numbers (Fig. 3B). To evaluate the variation range of (AG)_n_-Di-SSRs of the microbes with similar GC content, a regression equation with high R-value of 0.9936 was obtained, indicating the significant correlation between them. The variation range of (AG)_n_-Di-SSR was strictly limited when the GC % was too high or too low (Fig. 3C), which may explain the moderate counts of (AG)_n_-Di-SSRs in the three VP *Anaplasma* strains.

### Percentage ratio of (AG)_3_-Di-SSRs polymers

**Figure 4:**
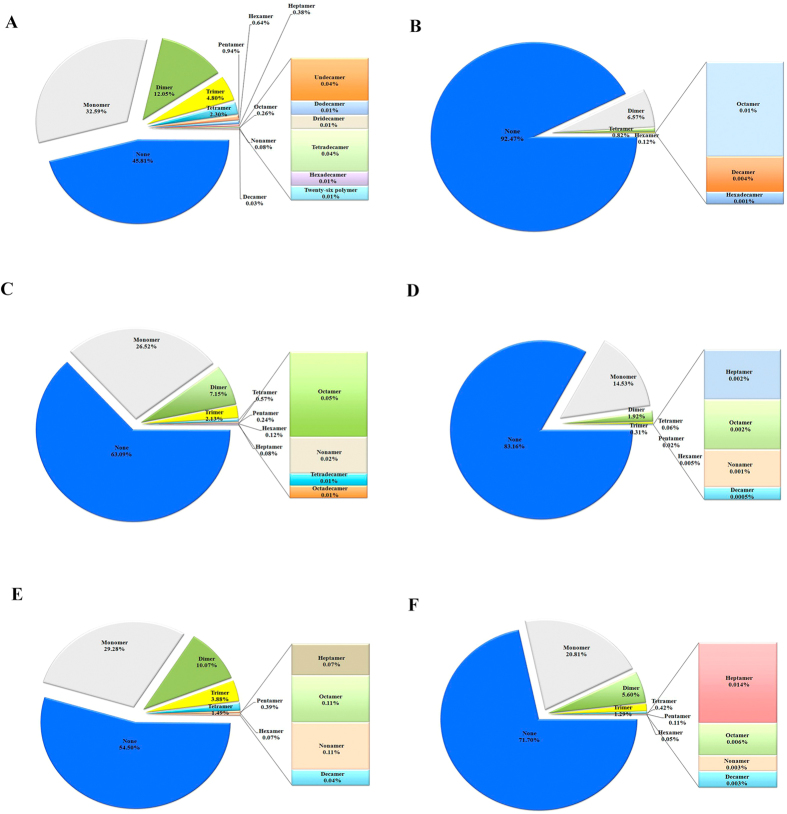
Pie charts showing the proportion of genes with different numbers of (AG)_3_-Di-SSRs per gene in *Mycoplasma*, *Rhizobiales* and *Rhickettsiales* genomes. **A** Vampire Pathogens (VP) of *Mycoplasma*; **B** Non Vampire Pathogens (N_VP) of *Mycoplasma*; **C** VP of *Rhizobiales*; **D** N_VP of *Rhizobiales*; **E** VP of *Rickettsiales*; **F** N_VP of *Rickettsiales*.

To explore the numbers of repeating units per gene, we focused the analysis on apparently conserved (AG)_3_-Di-SSRs, which presented overwhelming numbers in VP. Higher counts of genes with (AG)_3_-Di-SSRs was revealed for *Mycoplasma* VP, being totally absent genes in *Mycoplasma* N_VP (Fig. 4A-4B). We also found higher proportions of genes with three polymers of (AG)_3_-Di-SSRs in *Rhizobiales* and *Rickettsiales* VP, 7.0- and 3.0-fold greater than N_VP, respectively (Fig. 4C, 4D, 4E-4F). Retrieval of gene annotations from the genes with eight or more (AG)_3_-Di-SSRs in VP, what is considered extremely rare, revealed overrepresentation of genes related to nucleotide metabolism, bacterial surface as well as genes of unknown function (Supplementary Table S5).

Since the number of (AG)_3_-Di-SSRs per gene and gene sequence length may be proportional, relation between these two variables was investigated. Due to the large number of genes, the normality of sequence length and (AG)_3_-Di-SSRsnumber could not be assessed. Therefore, the “distribution independent” Spearman rank-correlation test was used to assess the correlation of these two variables. Although the results indicate that sequence length and (AG)_3_-Di-SSRs number were positively correlated (r = 0.36, *P* < 0.05), the low correlation coefficient suggested other factors influence (AG)_3_-Di-SSRs distribution, such as gene functions.

### Enriched GO terms analysis of genes carrying (AG)_3_-Di-SSRs

To determine which gene functions offer survival advantage for VP, GO terms were retrieved for all CDS and a subset of CDS containing 3 or more (AG)_3_-Di-SSR were taken as candidates to perform Fisher’s exact test against all CDS. Previous study found few statistically significant associations between SSRs in obligate pathogens and gene functional classifications^5^. However, in this study, plenty of significant GO terms (*FDR* < 0.01) were observed (Data not shown).

**Figure 5:**
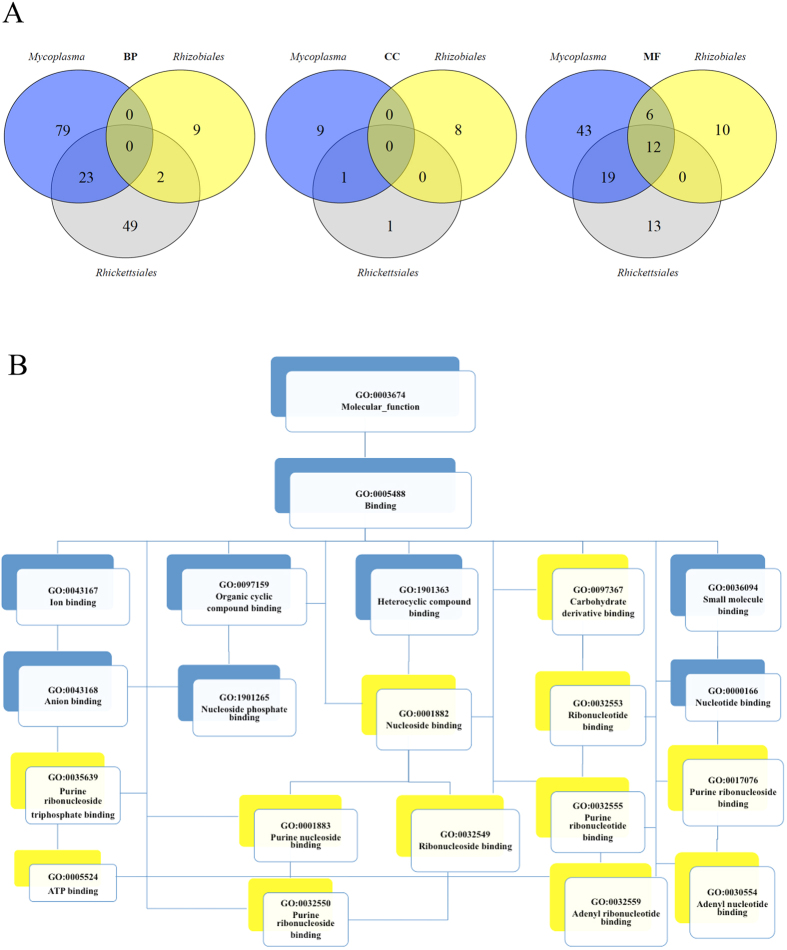
Enriched GO terms with (AG)_3_-Di-SSRs in Vampire Pathogens (VP) of *Mycoplasma*, *Rhizobiales* and *Rhickettsiales*. **A** Venn diagrams for enriched GO terms in biological process (BP), cellular component (CC) and molecular function (MF); **B** Directed acyclic graph of the common enriched MF GO terms in VP; the 12 shared GO terms in VP are indicated in yellow boxes.

With regard to *Mycoplasma* and *Rickettsiale*s, both groups followed similar enriched GO terms among the three Gene Ontologies, while *Rhizobiales* were more distant (Fig. 5A). However, an important overlap among the GO terms belonging to molecular function (MF) ontology was observed, with 12 GO terms, belonging to purine ribonucleoside triphosphate binding MFs (Data not shown). Identification of enriched GO terms with abundance of (AG)_3_-Di-SSRs shared by the VP of *Mycoplasma*, *Rhizobiales* and *Rickettsiale*s was critical for understanding the evolution of VP, suggesting that purine metabolism and transport may be important for microbial growth in bloodstream, where purine and nucleotide precursors are severely limited^1^. Venn diagrams revealed the overlap of 12 different GO terms common to all VP (Fig. 5A), which were represented in a directed acyclic tree generated using RamiGO R package (Fig. 5B). The lowest nodes belonged to ATP binding. We noted that higher nodes showed molecular functions involved in purine nucleotide and nucleoside binding.

**Figure 6:**
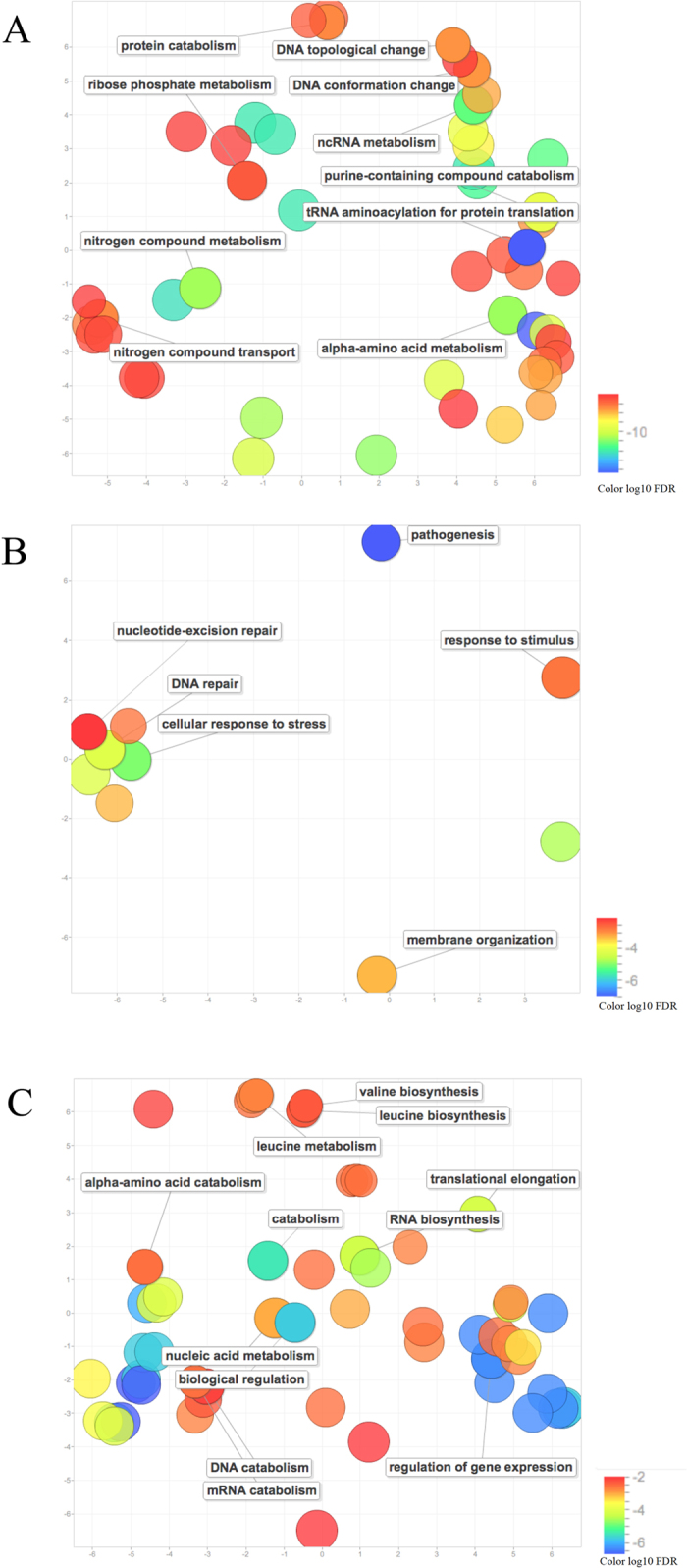
GO enrichment of genes with 3 or more (AG)_3_-Di-SSRs per gene. **A** scatterplot of enriched GO terms in *Mycoplasma*; **B** scatterplot of enriched GO terms in *Rhizobiales*; **C** scatterplot of enriched GO terms in *Rhickettsiales*.

Important overlap was found among the enriched BPs of *Mycoplasma* and *Rickettsiale*s. The corresponding genes belonged to glycosyl, nucleic acid and RNA metabolic process. GO terms particular for *Mycoplasma* were amino acid metabolic process and transmembrane transport, as well as DNA topological change and nitrogen compound transport. GO terms particular for *Rhizobiales* were mainly related to membrane, with important representation of outer membrane and pathogenesis genes, with high statistical significance, thus suggesting an important virulence mechanism. Other category of GO terms was related to cellular response against DNA damage, such as nucleotide-excision repair components. In addition, regulation of translation was observed for *Rickettsiale*s. Considering the hierarchical organization of GO terms, visualization of the enriched GO terms related to BP was facilitated using REVIGO online tool, revealing in a graphical manner the clusters of overrepresented GO terms (Fig. 6).

A key finding was that membrane-related genes were dramatically overrepresented among the genes with (AG)_3_-Di-SSRs. For instance, *Mycoplasma* presented active transmembrane transporter activity, with ATP-binding cassette (ABC) transporter complex as its main representative, required for iron acquisition and virulence in many pathogens^8^. Similar trend was observed for *Rhizobiales*, having outer membrane, membrane and pathogenesis overrepresented (Fig. 6B). Long SSRs composed of the oligonucleotides repeats of 1–4 bp length (LSSR^1^,^2^,^3^,^4^) in those genes were reported to contribute to immune evasion by enhancing antigenic variance^2^. The reduced amount of virulence genes found for *Mycoplasma* may be due to having only 26% of the genes with assigned GO terms, being 71–74% for the other two orders. However, it was interesting to note that 22% of CDS without GO term assignation were proteins with either signal peptide domain or transmembrane helices, suggesting many hypothetical proteins unavailable in the GO term analysis may be surface-associated (Data not shown).

### Analysis of Di-amino acid pairs

Although we assumed that some genes with (AG)_n_-Di-SSRs associated with purine nucleotide metabolism benefit VP in blood, it was important to note that random models of SSRs such as (AT)_n_, (AC)_n_ or (CG)_n_ Di-SSRs should also result in slipped strand mispairing and hypermutable loci subject to reversible changes. However, why only the (AG)_n_-Di-SSRs motif was abundant in VP? We explored the hints from genetic code and found significantly higher counts of Di-amino acids including Glu-Arg, Arg-Glu, and Leu-Ser coded by (AG)_3_, (GA)_3_, and (TC)_3_ in VP, but not Ser-Leu Di-amino acids encoded by (CT) (Data not shown). Di-amino acids coded by (AG)_n_-Di-SSRs included all three six-fold code amino acids (Arg, Leu and Ser), which may be related to the evolutionary importance of those amino acids, already present in the early genetic code and performing important balancing roles for error minimization by physicochemical properties^9^.

### Prediction of triplexes with (AG)_n_-Di-SSRs in *Mycoplasma*

It was not clear whether the role of (AG)_n_-Di-SSRs might be more important for gene function or for gene regulation, since the count of (AG)_n_-Di-SSRs in both coding and noncoding regions significantly differed between VP and N_VP (Supplementary Table S2). We explored the mechanism by which (AG)_n_-Di-SSR abundance modulates gene expression to benefit VP growth. (AG)_n_-Di-SSRs may contribute to the formation of a hinged DNA structure (H-DNA) by repeating copolymers of (dT-dC)_n_.(dA-dG)_n_^10^. Here we found significant differences (*P* < 0.001) on the numbers of triplexes formed from (AG)_n_-Di-SSRs between VP and N_VP by identification of potential intramolecular triplex-forming sequences (Supplementary Figure S1A). On the other hand, the count of all triplex types in VP had no quantitative advantage versus N_VP (Supplementary Figure S1B), even with the high proportion of triplexes associated with (AG)_n_-Di-SSRs (Supplementary Figure S1C).

### Global differentiation of SSRs profile in VP

As the basic element of (AG)_n_-Di-SSRs, the enrichment of AG dinucleotides may also influence the profiles of other types of SSRs. Thus, besides Di-SSRs, we also investigated from Mono- to Deca- SSRs in all 145 chromosomes. The longer the oligonucleotide is, the more kinds of the corresponding type of SSRs are. From all 145 chromosomes, we detected 222 kinds (33 subtypes) of Tetra-SSRs, 261 kinds (75 subtypes) of Penta-SSRs, 602 kinds (204 subtypes) of Hexa-SSRs, 134 kinds (95 subtypes) of Hepta-SSRs, 73 kinds (51 subtypes) of Octa-SSRs, 112 kinds (99 subtypes) of Nona-SSRs and 15 kinds (15 subtypes) of Deca-SSRs (Supplementary Table S6). Since the number of each subtype in each chromosome was low, it was inappropriate to perform statistic analysis.

**Figure 7:**
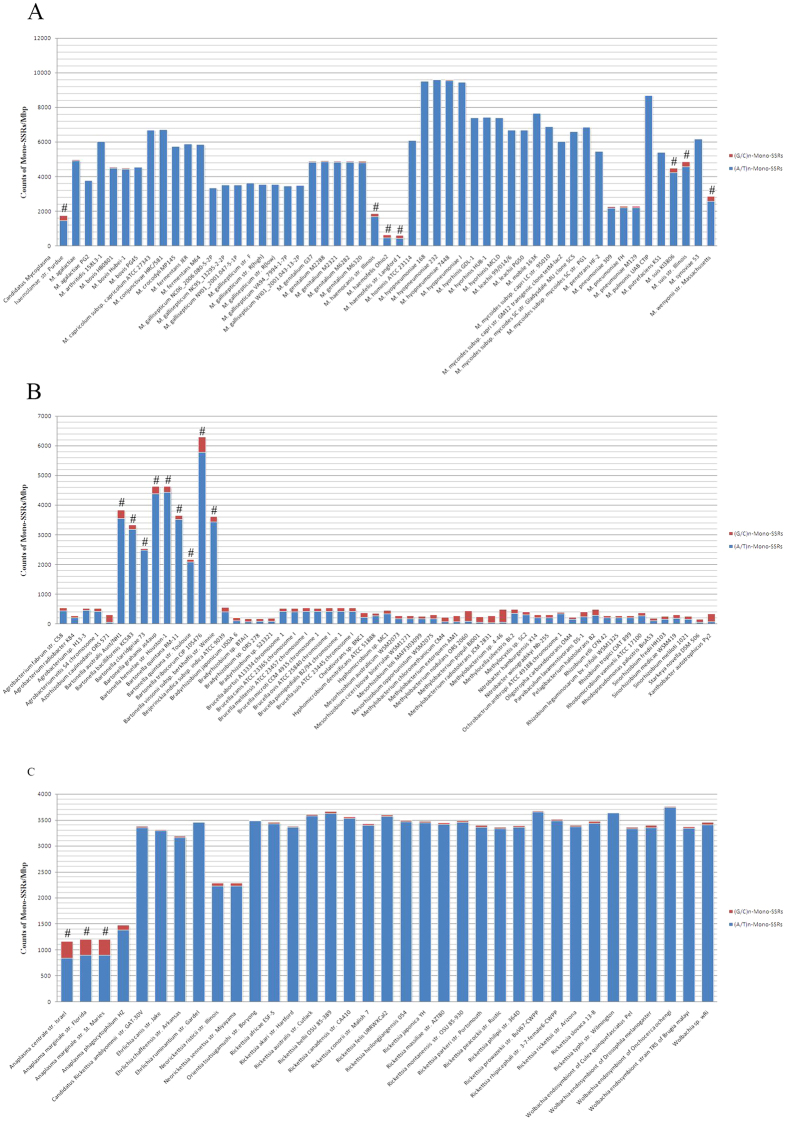
Density distribution of Mono-SSRs in the genomes of *Mycoplasma*, *Rhizobiales* and *Rhickettsiales*. **A** *Mycoplasma*; **B** *Rhizobiales* and **C** *Rickettsiales*. ^#^Vampire Pathogens (VP).

In terms of the profiles of Mono-SSRs and Tri-SSRs, there were some interesting differences between VP and N_VP. In *Rickettsiale*s the VP had more (G/C)_n_-Mono-SSRs (*P* = 0.005), while the number of (A/T)_n_-Mono-SSRs in VP of *Rhizobiales* was much higher (*P* < 0.001). In above cases, the effect of the distinct GC content of the VP compared to N_VP in these two orders might be involved (Fig. 7B,C). However, the abundance of (G/C)_n_-Mono-SSRs in VP of *Mycoplasma* was also higher than the N_VP while the abundance of (A/T)_n_-Mono-SSRs was smaller (*P* < 0.001), which might be due to the same factors as (AG)_n_-Di-SSRs (Fig. 7A).

**Figure 8:**
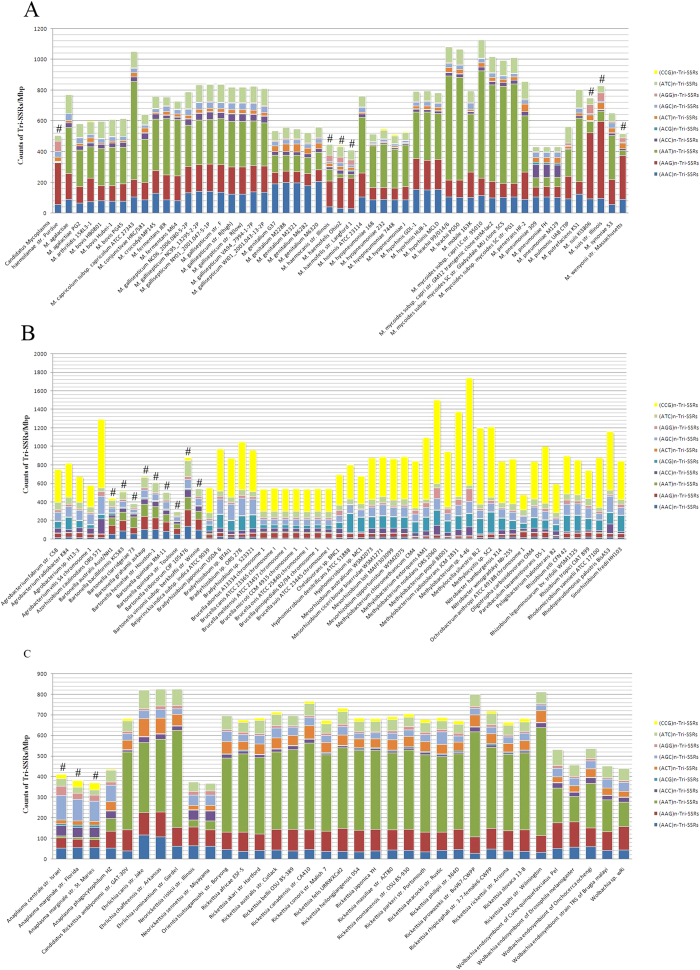
Density distribution of Tri-SSRs in the genomes of *Mycoplasma*, *Rhizobiales* and *Rhickettsiales*. **A** *Mycoplasma*; **B** *Rhizobiales* and **C** *Rickettsiales*. ^#^Vampire Pathogens (VP).

The abundance of (AAG)_n_-Tri-SSRs and (AGG)_n_-Tri-SSRs in VP of *Mycoplasma* was higher than the N_VP (*P* < 0.001). The VP of *Rickettsiale*s had more (AGC)_n_-Tri-SSRs and less (ATC)_n_-Tri-SSRs (*P* = 0.005) (Fig. 8A,C). In the VP of *Rhizobiales* there were more (AAC)_n_-Tri-SSRs, (AAG)_n_-Tri-SSRs and (AAT)_n_-Tri-SSRs while fewer (ACG)_n_-Tri-SSRs and (CCG)_n_-Tri-SSRs (*P* < 0.001) (Fig. 8B and Supplementary Table S7). Though in cases of Tri-SSRs the differences of the profiles between VP and N_VP were more complicated, as a part of repetitive unit, the potential influence of the AG dinucleotide was still notable.

Taken together, this study addressed (AG)_n_-Di-SSRs as a potential important survival code in the evolution of “Vampire Pathogens”.

## Methods

### Genome Sequences

A total of 145 complete prokaryotic chromosomes and coding nucleotide sequences were downloaded from the National Center for Biotechnology Information FTP server at ftp://ftp.ncbi.nih.gov/genomes/Bacteria/. For each prokaryotic strain, only one main replicon was analyzed (plasmids or extra smaller chromosomes were not included in the analysis).

Random genomes were created by Genome Randomizer (http://www.cmbl.uga.edu/software.html) with the m1c1 model, which generated intergenic regions with first-order Markov mode^11^. We compared the count of Di-SSRs per Mbp in 12 random and 12 real genomes of *Mycoplasma* by paired t-test (*P* < 0.05) (SigmaPlot 12.0; Fig. 1). *Mycoplasma* species having at least two fully sequenced strains were chosen from 53 complete sequences to obtain standard deviations (Supplementary Table S8).

### Simple Sequence Repeats

MISA (http://pgrc.ipkgatersleben.de/misa/misa.html), a program written in Perl, was utilized to generate the SSR data of all nucleotide sequences, including the numbers and length of repetitive units in all evaluated genomes^12^. The minimum length of an SSR was defined as 6 bp, setting three the minimum number of repeats of the corresponding oligonucleotide (repetitive unit). Considering the double-stranded structure of DNA, the SSRs from reverse-complement sequences were placed in the same category. The classification method was default in MISA which based on the shifting of the repetitive units. Taking (AGC)_3_-Tri-SSR as an example, three different repetitive units can be parsed from AGCAGCAGC, they are AGC, GCA and CAG. Therefore, (AGC)_n_, (GCA)_n_, (CAG)_n_ and their reverse-complement sequences were treated as a subtype. Here, the four subtypes of Di-SSRs were dimeric repeats of AC/CA/GT/TG, AG/GA/CT/TC, AT/TA and CG/GC, which were abbreviated (AC)_n_, (AG)_n_, (AT)_n_ and (CG)_n_, respectively. There were two subtypes (A/T, G/C) in Mono-SSR while Tri-SSRs had 10 subtypes. The count of SSRs in each genome, coding region and non-coding region were standardized to the length of genomes, coding regions and non-coding region, respectively (Figs 2,7 and 8).

### Correlation between Di-SSR and GC content

The correlation between the number of each subtype of Di-SSRs and GC content was analyzed by nonlinear regression with SigmaPlot 12.0. The R-value was used to evaluate the correlation (R > 0.9 indicated a good correlation) under the assumption that all parameters in the regression equation should pass the t-test (*P* < 0.0001) and the equation passed the significance test (F-test, *P* < 0.0001). To normalize the observations, we used the arcsine square root transformation by calculating the square root and arcsine of the value {ASIN [SQRT (Proportion)]} (Fig. 3B).

The impact of genome GC content was evaluated in relation with variation range of (AG)_n_-Di-SSR. All 145 strains were sorted in ascending order GC content, and the distance between the highest and lowest values was divided into 3% interval. Groups with less than 10 strains would be combined with the contiguous group(s) until they had more than 10 strains. Regression analysis was performed to evaluate the significant of correlation between the class midpoint as independent variable and the (AG)_n_-Di-SSR variation range of the strains within a class as dependent variable (Fig. 3C).

### Significant analyses of SSRs in VP

The (AG)_n_-Di-SSR Mono-SSR and Tri-SSR count of *Mycoplasma, Rhizobiales* and *Rickettsiales* strains, separated into VP group and N_VP group, were statistically compared in genomes, and for (AG)_n_-Di-SSR, coding regions and non-coding regions were also investigated separately. Rank sum test (Mann-Whitney) was used to assess significance (Supplementary Table S2, Supplementary Table S7).

(AG)_n_-Di-SSR in each VP were compared with other strains in the same taxonomic category, and differences were calculated with the cutoff value set by Chebyshev’s inequality. As the inequality stated, at least 1–1/k^2^ percent of the observations would lie within k standard deviations (s_x_) of the mean (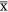), with k = √8 and k = 8 as cutoff values to evaluate significance. If the candidate value was above 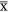 + √8 s_x_, it was considered remarkably deviated from the norm; the difference was treated as significant when value exceeded 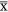 + 8 s_x_. All proportion data were transformed using {ASIN [SQRT (Proportion)]} before analysis (Supplementary Table S4).

### Analysis of the numbers of repeating units carried by each gene

Coding sequences from *Mycoplasma*, *Rhizobiales* and *Rickettsiales* strains were extracted from GenBank files using gb2tab (http://www.cbs.dtu.dk/services/FeatureExtract/download.php) and divided into VP and N_VP groups. The CDS from each group were scanned to retrieve the numbers of (AG)_3_-Di-SSRs per CDS by using MISA (Fig. 4 and Supplementary Table S5).

### GO term enrichment

CDS in the VP of *Mycoplasma, Rhizobiales* and *Rickettsiales* were blasted using BLASTp (version 2.2.28) against a reduced non-redundant NCBI database (September 2013). GO terms were retrieved using Blast2GO^13^, as well as additional functional annotations such as transmembrane helices and signal peptides. CDS containing more than three (AG)_3_-Di-SSRs were identified as candidates for classic Fisher’s exact testing against all CDS through BLAST2GO built-in tool^13^. Tests were performed for biological process (BP), cellular component (CC) and molecular function (MF) with *FDR* < 0.01 (Fig. 5). Significant GO terms shared by the VP of *Mycoplasma, Rhizobiales* and *Rickettsiales* were compared using BioVenn^14^ (Fig. 5A). Shared GO terms were visualized with RamiGO^15^ R package (Fig. 5B), and further interpretation was facilitated using REVIGO online tool^16^.

### Analysis of Di-amino acid pairs

We calculated the numbers of (AG)_3_-Di-SSRs in frame with each ORF in coding regions by the location of each (AG)_3_-Di-SSRs, which was obtained from the Location File created by Misa.pl, and retrieved the Protein File of each strain to count the total numbers of each kind of Di-amino acid pairs (ER/RE/SL/LS). We determined the in-frame (AG)_3_-Di-SSRs as the number of Di-amino acid pairs, and then obtained the proportion of Di-amino acid pairs.

### Analyses of Triplexes

We used the R/Bio conductor package “Triplex” with default parameters (min_score = 17; min_len = 8) to identify the potential triplex patterns in *Mycoplasma* genomes^17^ (Supplementary Figure S1). All identified triplexes and the nucleotide sequences capable of forming H-DNA were divided into categories with or without (AG)_n_-Di-SSRs. Then the proportion of the triplexes with (AG)_n_-Di-SSRs in each genome was calculated and compared in VP with N_VP group using Mann-Whitney Rank sum test (*P* < 0.001) after data transformation.

## Additional Information

**How to cite this article**: Zou, G. *et al.* The Cipher Code of Simple Sequence Repeats in “Vampire Pathogens”. *Sci. Rep.* **5**, 12441; doi: 10.1038/srep12441 (2015).

## Supplementary Material

Supplementary Information
