# Corrigendum: The Cipher Code of Simple Sequence Repeats in “Vampire Pathogens”

**DOI:** 10.1038/srep31530

**Published:** 2016-08-23

**Authors:** Geng Zou, Bernardo Bello-Orti, Virginia Aragon, Alexander W. Tucker, Rui Luo, Pinxing Ren, Dingren Bi, Rui Zhou, Hui Jin

Scientific Reports 5: Article number: 1244110.1038/srep12441; published online: 07 28 2015; updated: 08 23 2016

In this Article, there is a typographical error in the Acknowledgments section.

“This work was supported by grants from National Natural Science Foundation of China (NSFC, grant no. 31201931), National Basic Research Program (973 Program, grant no. 2012CB18802), FPI fellowship from the Spanish Ministerio de Economía y Competitividad (Grant no. BES-2011-047557) and China-UK Cooperation Programme in Global Priorities (CUKPGP, grant no. 2013DFG32360)”.

should read:

“This work was supported by grants from National Natural Science Foundation of China (NSFC, grant no. 31201931), National Basic Research Program (973 Program, grant no. 2012CB518802), FPI fellowship from the Spanish Ministerio de Economía y Competitividad (Grant no. BES-2011-047557) and China-UK Cooperation Programme in Global Priorities (CUKPGP, grant no. 2013DFG32360)”.

