# Supplementary material for: The Cipher Code of Simple Sequence Repeats in “Vampire Pathogens”
Source: Sci Rep. 2015 Jul 28;5:12441. doi: 10.1038/srep12441 (PMC4516964; doi:10.1038/srep12441)
Supplement: Supplementary Information [file srep12441-s1.doc]

**Supplementary Information**

**The Cipher Code of Simple Sequence Repeats in “Vampire Pathogens”**

Geng Zou^1,*^, Bernardo Bello-Orti^2,*^, Virginia Aragon^2,3^, Alexander W. Tucker^4^, Rui Luo^1^, Pinxing Ren^1^, Dingren Bi^1^, Rui Zhou^1^& Hui Jin^1^

^1^State Key Laboratory of Agricultural Microbiology, College of Veterinary Medicine, Huazhong Agricultural University, Wuhan 430070, China,^2^Centre de Recerca en Sanitat Animal (CReSA), UAB-IRTA, Campus de la Universitat Autònoma de Barcelona, 08193 Bellaterra, Spain,^3^Institut de Recerca i Tecnologia Agroalimentàries (IRTA), Barcelona, Spain,^4^Department of Veterinary Medicine, University of Cambridge, Cambridge, United Kingdom.

^*^ These authors contributed equally to this work.

Corresponding Author: Hui Jin(E-mail: [jinhui@mail.hzau.edu.cn](mailto:jinhui@mail.hzau.edu.cn)).


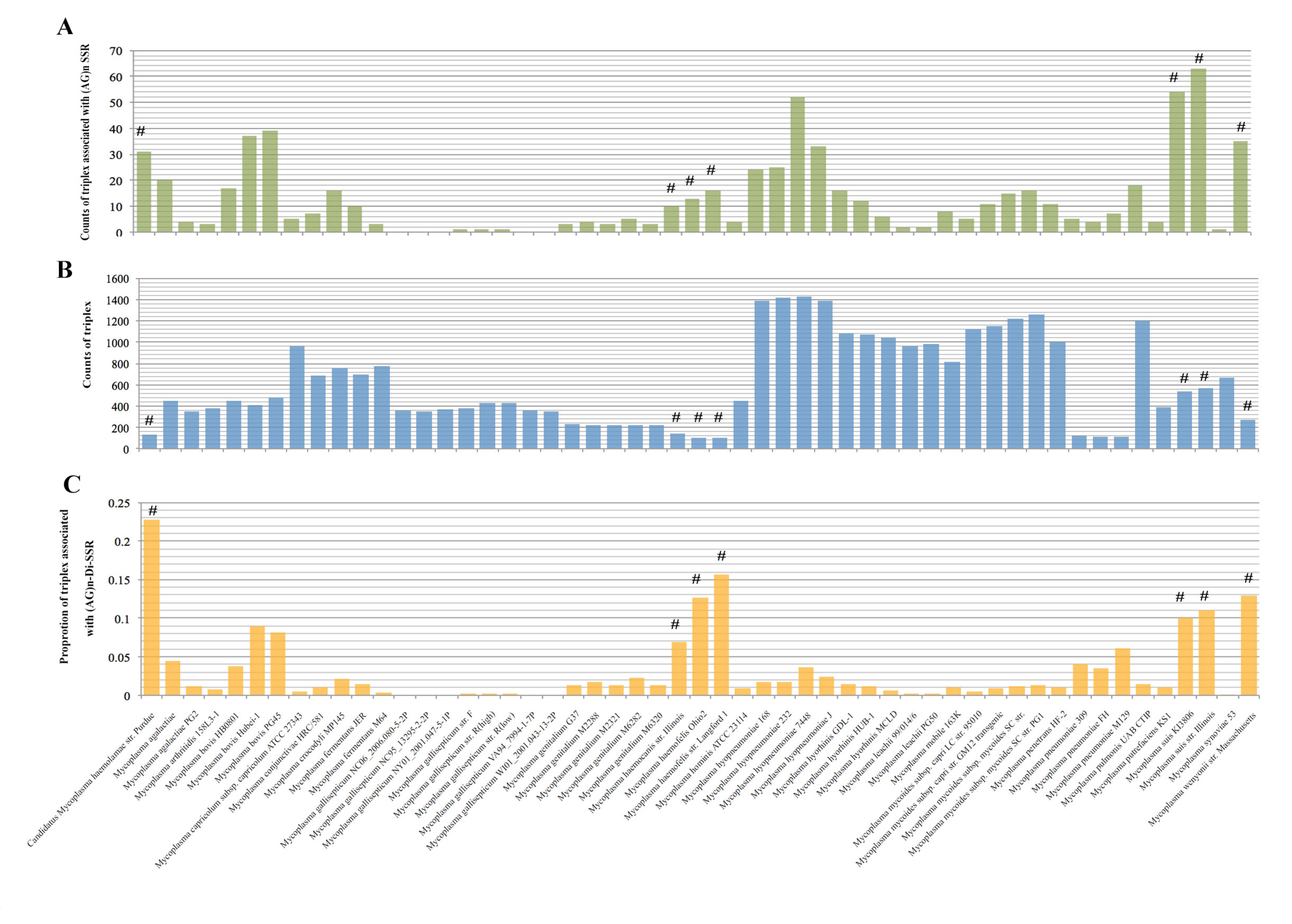


**Supplementary Figure S1 |Prediction of triplexes with (AG)_n_-Di-SSRs in *Mycoplasma***A, Triplexes associated with (AG)_n_-Di-SSRs; B, All triplex subtypes; C, Proportion of triplexes associated with (AG)_n_-Di-SSRs. ^#^ Vampire Pathogens (VP).

**Supplementary Table S1. The characteristic of the Vampire Pathogens** strains

| **Name of Strains** | **Host** | **Isolation Site Recorded** | **GC %**  **(%)** | **Genome**  **length (bp)** | **Classification** |
| --- | --- | --- | --- | --- | --- |
|  |  |  |  |  |  |
| *Mycoplasma wenyonii* str. Massachusetts | Bos taurus | Blood sample of a cow | 33.9 | 650228 | *Tenericutes/Mollicutes/Mycoplasmatales/Mycoplasmataceae/Mycoplasma* |
| *Mycoplasma haemocanis* str. Illinois | Canis familiaris | Blood of naturally infected dog | 35.3 | 919992 | *Tenericutes/Mollicutes/Mycoplasmatales/Mycoplasmataceae/Mycoplasma* |
| *Mycoplasma suis* KI3806 | Sus scrofa | Blood of an infected pig | 31.1 | 70927 | *Tenericutes/Mollicutes/Mycoplasmatales/Mycoplasmataceae/Mycoplasma* |
| *Mycoplasma haemofelis* str. Langford 1 | Felis catus | Blood of an experimentally infected cat | 38.9 | 1147259 | *Tenericutes/Mollicutes/Mycoplasmatales/Mycoplasmataceae/Mycoplasma* |
| *Mycoplasma haemofelis* Ohio2 | Felis catus | Blood of an experimentally infected cat | 38.8 | 1155937 | *Tenericutes/Mollicutes/Mycoplasmatales/Mycoplasmataceae/Mycoplasma* |
| *Mycoplasma suis* str. Illinois | Sus scrofa | Blood of an infected pig | 31.1 | 742431 | *Tenericutes/Mollicutes/Mycoplasmatales/Mycoplasmataceae/Mycoplasma* |
| *Candidatus Mycoplasma haemolamae* str. Purdue | Alpaca | Alpaca | 39.3 | 756845 | *Tenericutes/Mollicutes/Mycoplasmatales/Mycoplasmataceae/Mycoplasma* |
| *Bartonella vinsonii subsp. berkhoffii str. Winnie* | Dog | Pekingese | 38.8 | 1802699 | *Proteobacteria/Alphaproteobacteria/Rhizobiales/Bartonellaceae/Bartonella* |
| *Bartonella tribocorum* CIP 105476 | Rat | Blood of two wild rats | 38.9 | 2619061 | *Proteobacteria/Alphaproteobacteria/Rhizobiales/Bartonellaceae/Bartonella* |
| *Bartonella quintana* str. Toulouse | Homo sapiens | NA | 38.8 | 1581384 | *Proteobacteria/Alphaproteobacteria/Rhizobiales/Bartonellaceae/Bartonella* |
| *Bartonella quintana* RM-11 | Rhesus macaque | Rhesus macaque | 38.8 | 1587646 | *Proteobacteria/Alphaproteobacteria/Rhizobiales/Bartonellaceae/Bartonella* |
| *Bartonella henselae* str. Houston-1 | Felis catus and Homo sapiens | NA | 38.2 | 1931047 | *Proteobacteria/Alphaproteobacteria/Rhizobiales/Bartonellaceae/Bartonella* |
| *Bartonella grahamii* as4aup | Rat | Rat | 38.1 | 2341328 | *Proteobacteria/Alphaproteobacteria/Rhizobiales/Bartonellaceae/Bartonella* |
| *Bartonella clarridgeiae* 73 | Felis catus and Canis familiaris | Scratch of cats | 35.7 | 1522743 | *Proteobacteria/Alphaproteobacteria/Rhizobiales/Bartonellaceae/Bartonella* |
| *Bartonella bacilliformis* KC583 | Homo sapiens | NA | 38.2 | 1445021 | *Proteobacteria/Alphaproteobacteria/Rhizobiales/Bartonellaceae/Bartonella* |
| *Bartonella australis* Aust/NH1 | Macropus giganteus | Blood of Macropus Giganteus (gray kangaroos) | 41.8 | 1596490 | *Proteobacteria/Alphaproteobacteria/Rhizobiales/Bartonellaceae/Bartonella* |
| *Anaplasma marginale* str. St. Maries | Cow | Acutely infected cow | 49.8 | 1197687 | *Proteobacteria/Alphaproteobacteria/Rickettsiales/Anaplasmataceae/Anaplasma* |
| *Anaplasma marginale* str. Florida | Cow and Homo sapiens | Acutely infected cow | 49.8 | 1202435 | *Proteobacteria/Alphaproteobacteria/Rickettsiales/Anaplasmataceae/Anaplasma* |
| *Anaplasma centrale* str. Israel | Cow | NA | 50 | 1206806 | *Proteobacteria/Alphaproteobacteria/Rickettsiales/Anaplasmataceae/Anaplasma* |

NA: not available.

**Supplementary Table S2. Significance assessments of the count of (AG)_n_-Di-SSRs between Vampire Pathogens (VP) and non Vampire Pathogens (N_VP) with Mann-Whitney Rank Sum Test**

| **Scopes** | **Group** | **Numbers of Genomes** | **Median** | **25%** | **75%** | | **Mann-Whitney U Statistic** | | **T** | | **P value** | |
| --- | --- | --- | --- | --- | --- | --- | --- | --- | --- | --- | --- | --- |
| Genomes | VP | 19 | 850.39 | 731.41 | | 1276.89 | | 53.00 | | 2531.00 | | *** |
|  | N_VP | 126 | 280.42 | 182.88 | | 390.390 | |  |  |  |  |  |
| Coding Regions | VP | 19 | 728.50 | 610.12 | | 1332.55 | | 76.00 | | 2508.00 | | *** |
|  | N_VP | 126 | 239.68 | 164.54 | | 367.166 | |  |  |  |  |  |
| Non-Coding Regions | VP | 19 | 1085.01 | 772.93 | | 1359.10 | | 60.00 | | 2524.00 | | *** |
|  | N_VP | 126 | 424.15 | 285.58 | | 627.73 | |  |  |  |  |  |
| Genomes of *Mycoplasma* | VP | 7 | 1405.79 | 1273.14 | | 2256.74 | | 0.00 | | 350.00 | | *** |
|  | N_VP | 46 | 169.50 | 140.93 | | 245.11 | |  |  |  |  |  |
| Coding Regions of *Mycoplasma* | VP | 7 | 1432.98 | 1298.08 | | 2305.46 | | 0.00 | | 350.00 | | *** |
|  | N_VP | 46 | 163.63 | 121.05 | | 230.87 | |  |  |  |  |  |
| Non-Coding Regions of *Mycoplasma* | VP | 7 | 1085.01 | 819.46 | | 1630.17 | | 0.00 | | 350.00 | | *** |
|  | N_VP | 46 | 266.22 | 195.16 | | 358.29 | |  |  |  |  |  |
| Genomes of *Rhizobiales* | VP | 9 | 784.18 | 629.77 | | 871.92 | | 0.00 | | 477.00 | | *** |
|  | N_VP | 48 | 275.80 | 232.50 | | 336.24 | |  |  |  |  |  |
| Coding Regions of *Rhizobiales* | VP | 9 | 610.12 | 557.30 | | 702.77 | | 1.00 | | 476.00 | | *** |
|  | N_VP | 48 | 216.26 | 176.62 | | 284.86 | |  |  |  |  |  |
| Non-Coding Regions of *Rhizobiales* | VP | 9 | 1233.76 | 1017.82 | | 1370.60 | | 8.00 | | 469.00 | | *** |
|  | N_VP | 48 | 631.48 | 538.44 | | 700.65 | |  |  |  |  |  |
| Genomes of *Rickettsiales* | VP | 3 | 731.41 | 722.70 | | 734.17 | | 12.00 | | 90.00 | | * |
|  | N_VP | 32 | 417.99 | 401.97 | | 573.07 | |  |  |  |  |  |
| Coding Regions of *Rickettsiales* | VP | 3 | 728.50 | 718.61 | | 737.91 | | 13.00 | | 89.00 | | * |
|  | N_VP | 32 | 423.37 | 394.83 | | 598.39 | |  |  |  |  |  |
| Non-Coding Regions of *Rickettsiales* | VP | 3 | 747.08 | 714.22 | | 748.71 | | 6.00 | | 96.00 | | * |
|  | N_VP | 32 | 404.81 | 379.27 | | 534.66 | |  |  |  |  |  |

The difference in the median values between the two groups is greater than would be expected by chance; there is a statistically significant difference (* 0.01 < P < 0.05, *** P < 0.001).

**Supplementary Table S3. The repeating units numbers of (AG)_n_-Di-SSRs in Vampire Pathogens** genomes

| **#** | **Name of Strains** | **Counts of (AG)_n_-Di-SSR Repeats** | | | | | | | | | | |
| --- | --- | --- | --- | --- | --- | --- | --- | --- | --- | --- | --- | --- |
|  |  | 3 | 4 | 5 | 6 | 7 | 8 | 9 | 10 | 11 | 12 | 13 |
| 1 | *Anaplasma centrale* str. Israel | 714 | 32 | 3 | 1 | 0 | 0 | 0 | 0 | 0 | 0 | 0 |
| 2 | *Anaplasma marginale* str. Florida | 696 | 39 | 4 | 1 | 0 | 0 | 0 | 0 | 0 | 0 | 0 |
| 3 | *Anaplasma marginale* str. St. Maries | 703 | 39 | 4 | 1 | 0 | 0 | 0 | 0 | 0 | 0 | 0 |
| 4 | *Bartonella bacilliformis* KC583 | 607 | 54 | 2 | 0 | 0 | 0 | 0 | 0 | 0 | 0 | 0 |
| 5 | *Bartonella clarridgeiae* 73 | 530 | 37 | 4 | 0 | 0 | 0 | 0 | 0 | 0 | 0 | 0 |
| 6 | *Bartonella grahamii* as4aup | 1027 | 98 | 8 | 0 | 0 | 0 | 0 | 0 | 0 | 0 | 0 |
| 7 | *Bartonella henselae* str. Houston-1 | 931 | 91 | 9 | 2 | 0 | 0 | 0 | 0 | 0 | 0 | 0 |
| 8 | *Bartonella quintana* RM-11 | 643 | 56 | 4 | 0 | 0 | 0 | 0 | 0 | 0 | 0 | 0 |
| 9 | *Bartonella quintana* str. Toulouse | 636 | 55 | 5 | 0 | 0 | 0 | 0 | 0 | 0 | 0 | 0 |
| 10 | *Bartonella tribocorum* CIP 105476 | 1246 | 127 | 15 | 0 | 0 | 0 | 0 | 0 | 0 | 0 | 0 |
| 11 | *Bartonella australis* Aust/NH1 | 638 | 46 | 3 | 2 | 0 | 0 | 0 | 0 | 0 | 0 | 0 |
| 12 | *Bartonella vinsonii* subsp. berkhoffii str. Winnie | 859 | 65 | 9 | 1 | 0 | 0 | 0 | 0 | 0 | 0 | 0 |
| 13 | *Candidatus Mycoplasma haemolamae* str. Purdue | 1359 | 131 | 7 | 1 | 0 | 0 | 0 | 0 | 0 | 0 | 0 |
| 14 | *Mycoplasma suis* KI3806 | 755 | 44 | 3 | 1 | 0 | 0 | 0 | 1 | 0 | 0 | 0 |
| 15 | *Mycoplasma suis* str. Illinois | 830 | 44 | 3 | 0 | 1 | 0 | 0 | 0 | 2 | 1 | 1 |
| 16 | *Mycoplasma wenyonii str. Massachusetts* | 1273 | 141 | 12 | 0 | 0 | 0 | 0 | 0 | 0 | 0 | 0 |
| 17 | *Mycoplasma haemofelis* Ohio2 | 1436 | 88 | 2 | 1 | 0 | 0 | 0 | 0 | 0 | 0 | 0 |
| 18 | *Mycoplasma haemofelis* str. Langford 1 | 1494 | 84 | 1 | 0 | 0 | 0 | 0 | 0 | 0 | 0 | 0 |
| 19 | *Mycoplasma haemocanis* str. Illinois | 870 | 54 | 2 | 0 | 0 | 0 | 0 | 0 | 0 | 0 | 0 |

**Supplementary Table S4. Significant analysis of the count of (AG)_n_-Di-SSRs between Vampire Pathogens (VP) and non Vampire Pathogens (N_VP) with Chebyshev’s inequality**

| **#** | **Name of Strains** | **(AG)_n_ –Di-SSR counts** | | |
| --- | --- | --- | --- | --- |
|  |  | Genome | Coding Region | Non-Coding Region |
| **VP** | | | | |
| **1** | *Mycoplasma wenyonii* str. Massachusetts | ** | ** | ** |
| **2** | *Mycoplasma suis str. Illinois* | ** | ** | * |
| **3** | *Mycoplasma suis* KI3806 | ** | ** | * |
| **4** | *Mycoplasma haemofelis* str. Langford 1 | ** | ** | * |
| **5** | *Mycoplasma haemofelis* Ohio2 | ** | ** | * |
| **6** | *Mycoplasma haemocanis* str. Illinois | ** | ** | * |
| **7** | *Candidatus Mycoplasma haemolamae* str. Purdue | ** | ** | ** |
| **8** | *Bartonella vinsonii subsp. berkhoffii* str. Winnie | * | * | * |
| **9** | *Bartonella tribocorum* CIP 105476 | * | * | * |
| **10** | *Bartonella quintana* str. Toulouse | * | * | * |
| **11** | *Bartonella quintana* RM-11 | * | * | * |
| **12** | *Bartonella henselae* str. Houston-1 | * | * | * |
| **13** | *Bartonella grahamii* as4aup | * | * | * |
| **14** | *Bartonella clarridgeiae* 73 | * | * | - |
| **15** | *Bartonella bacilliformis* KC583 | * | * | * |
| **16** | *Bartonella australis* Aust/NH1 | * | * | - |
| **17** | *Anaplasma marginale* str. St. Maries | - | - | - |
| **18** | *Anaplasma marginale* str. Florida | - | - | - |
| **19** | *Anaplasma centrale* str. Israel | - | - | - |
| **N_VP in *Mycoplasma*** | | | | |
| **20** | *Mycoplasma agalactiae* | - | - | - |
| **21** | *Mycoplasma agalactiae* PG2 | - | - | - |
| **22** | *Mycoplasma arthritidis* 158L3-*1* | - | - | - |
| **23** | *Mycoplasma bovis* HB0801 | - | - | - |
| **24** | *Mycoplasma bovis* Hubei-1 | - | - | - |
| **25** | *Mycoplasma bovis* PG45 | - | - | - |
| **26** | *Mycoplasma capricolum* subsp. capricolum ATCC 27343 | - | - | - |
| **27** | *Mycoplasma conjunctivae* HRC/581 | - | - | - |
| **28** | *Mycoplasma crocodyli* MP145 | - | - | - |
| **29** | *Mycoplasma fermentans* JER | - | - | - |
| **30** | *Mycoplasma fermentans* M64 | - | - | - |
| **31** | *Mycoplasma gallisepticum* NC06_2006.080-5-2P | - | - | - |
| **32** | *Mycoplasma gallisepticum* NC95_13295-2-2P | - | - | - |
| **33** | *Mycoplasma gallisepticum* NY01_2001.047-5-1P | - | - | - |
| **34** | *Mycoplasma gallisepticum* str. F | - | - | - |
| **35** | *Mycoplasma gallisepticum* str. R (high) | - | - | - |
| **36** | *Mycoplasma gallisepticum* str.R (low) | - | - | - |
| **37** | *Mycoplasma gallisepticum* VA94_7994-1-7P | - | - | - |
| **38** | *Mycoplasma gallisepticum* WI01_2001.043-13-2P | - | - | - |
| **39** | *Mycoplasma genitalium* G37 | - | - | - |
| **40** | *Mycoplasma genitalium* M2288 | - | - | - |
| **41** | *Mycoplasma genitalium* M2321 | - | - | - |
| **42** | *Mycoplasma genitalium* M6282 | - | - | - |
| **43** | *Mycoplasma genitalium* M6320 | - | - | - |
| **44** | *Mycoplasma hominis* ATCC 23114 | - | - | - |
| **45** | *Mycoplasma hyopneumoniae* 168 | - | - | - |
| **46** | *Mycoplasma hyopneumoniae* 232 | - | - | - |
| **47** | *Mycoplasma hyopneumoniae* 7448 | - | - | - |
| **48** | *Mycoplasma hyopneumoniae* J | - | - | - |
| **49** | *Mycoplasma hyorhinis* GDL-1 | - | - | - |
| **50** | *Mycoplasma hyorhinis* HUB-1 | - | - | - |
| **51** | *Mycoplasma hyorhinis* MCLD | - | - | - |
| **52** | *Mycoplasma leachii* 99/014/6 | - | - | - |
| **53** | *Mycoplasma leachii* PG50 | - | - | - |
| **54** | *Mycoplasma mobile* 163K | - | - | - |
| **55** | *Mycoplasma mycoides* subsp. capri LC str. 95010 | - | - | - |
| **56** | *Mycoplasma mycoides* subsp. capri str. GM12 transgenic clone tetM-lacZ | - | - | - |
| **57** | *Mycoplasma mycoides* subsp. mycoides SC str. Gladysdale MU clone SC5 | - | - | - |
| **58** | *Mycoplasma mycoides* subsp. mycoides SC str. PG1 | - | - | - |
| **59** | *Mycoplasma penetrans* HF-2 | - | - | - |
| **60** | *Mycoplasma pneumoniae* 309 | - | - | - |
| **61** | *Mycoplasma pneumoniae* FH | - | - | - |
| **62** | *Mycoplasma pneumoniae* M129 | - | - | - |
| **63** | *Mycoplasma pulmonis* UAB CTIP | - | - | - |
| **64** | *Mycoplasma putrefaciens* KS1 | - | - | - |
| ***65*** | *Mycoplasma synoviae* 53 | - | - | - |
| **N_VP in *Rhizobiales*** | | | | |
| **66** | *Agrobacterium fabrum* str. C58 | - | - | - |
| **67** | *Agrobacterium radiobacter* K84 | - | - | - |
| **68** | *Agrobacterium sp.* H13-3 | - | - | - |
| **69** | *Agrobacterium vitis* S4 chromosome 1 | - | - | - |
| **70** | *Azorhizobium caulinodans* ORS 571 | - | - | - |
| **71** | *Beijerinckia indica subsp. indica* ATCC 9039 | * | * | - |
| **72** | *Bradyrhizobium japonicum* USDA 6 | - | - | - |
| **73** | *Bradyrhizobium sp.* BTAi1 | - | - | - |
| **74** | *Bradyrhizobium sp.* ORS 278 | - | - | - |
| **75** | *Bradyrhizobium sp.* S23321 | - | - | - |
| **76** | *Brucella abortus* A13334 chromosome 1 | - | - | - |
| **77** | *Brucella canis* ATCC 23365 chromosome I | - | - | - |
| **78** | *Brucella melitensis* ATCC 23457 chromosome I | - | - | - |
| **79** | *Brucella microti* CCM 4915 chromosome 1 | - | - | - |
| **80** | *Brucella ovis* ATCC 25840 chromosome I | - | - | - |
| **81** | *Brucella pinnipedialis* B2/94 chromosome 1 | - | - | - |
| **82** | *Brucella suis* ATCC 23445 chromosome I | - | - | - |
| **83** | *Chelativorans sp.* BNC1 | - | - | - |
| **84** | *Hyphomicrobium denitrificans* ATCC 51888 | - | - | - |
| **85** | *Hyphomicrobium sp.* MC1 | - | - | - |
| **86** | *Mesorhizobium australicum* WSM2073 | - | - | - |
| **87** | *Mesorhizobium ciceri* biovar biserrulae WSM1271 | - | - | - |
| **88** | *Mesorhizobium loti* MAFF303099 | - | - | - |
| **89** | *Mesorhizobium opportunistum* WSM2075 | - | - | - |
| **90** | *Methylobacterium chloromethanicum* CM4 | - | - | - |
| **91** | *Methylobacterium extorquens* AM1 | - | - | - |
| **92** | *Methylobacterium nodulans* ORS 2060 | - | - | - |
| **93** | *Methylobacterium populi* BJ001 | - | - | - |
| **94** | *Methylobacterium radiotolerans* JCM 2831 | - | - | - |
| **95** | *Methylobacterium sp.* 4-46 | - | - | - |
| **96** | *Methylocella silvestris* BL2 | - | - | - |
| **97** | *Methylocystis sp.* SC2 | - | - | - |
| **98** | *Nitrobacter hamburgensis* X14 | - | - | - |
| **99** | *Nitrobacter winogradskyi* Nb-255 | - | - | - |
| **100** | *Ochrobactrum anthropi* ATCC 49188 chromosome 1 | - | - | - |
| **101** | *Oligotropha carboxidovorans* OM4 | - | - | - |
| **102** | *Parvibaculum lavamentivorans* DS-1 | - | - | * |
| **103** | *Pelagibacterium halotolerans* B2 | - | - | - |
| **104** | *Rhizobium etli* CFN 42 | - | - | - |
| **105** | *Rhizobium leguminosarum* bv. trifolii WSM1325 | - | - | - |
| **106** | *Rhizobium tropici* CIAT 899 | - | - | - |
| **107** | *Rhodomicrobium vannielii* ATCC 17100 | - | - | - |
| **108** | *Rhodopseudomonas palustris* BisA53 | - | - | - |
| **109** | *Sinorhizobium fredii* HH103 | - | - | - |
| **110** | *Sinorhizobium medicae* WSM419 | - | - | - |
| **111** | *Sinorhizobium meliloti* 1021 | - | - | - |
| **112** | *Starkeya novella* DSM 506 | - | - | - |
| **113** | *Xanthobacter autotrophicus* Py2 | - | - | - |
| **N_VP in *Rickettsiales*** | | | | |
| **114** | *Anaplasma phagocytophilum* HZ | - | - | - |
| **115** | *Candidatus Rickettsia* amblyommii str. GAT-30V | - | - | - |
| **116** | *Ehrlichia canis* str. Jake | - | - | - |
| **117** | *Ehrlichia chaffeensis* str. Arkansas | - | - | - |
| **118** | *Ehrlichia ruminantium* str. Gardel | - | - | - |
| **119** | *Neorickettsia risticii* str. Illinois | - | - | - |
| **120** | *Neorickettsia sennetsu* str. Miyayama | - | - | - |
| **121** | *Orientia tsutsugamushi* str. Boryong | - | - | - |
| **122** | *Rickettsia africae* ESF-5 | - | - | - |
| **123** | *Rickettsia akari* str. Hartford | - | - | - |
| **124** | *Rickettsia australis* str. Cutlack | - | - | - |
| **125** | *Rickettsia bellii* OSU 85-389 | - | - | - |
| **126** | *Rickettsia canadensis* str. CA410 | - | - | - |
| **127** | *Rickettsia conorii* str. Malish 7 | - | - | - |
| **128** | *Rickettsia felis* URRWXCal2 | - | - | - |
| **129** | *Rickettsia heilongjiangensis* 054 | - | - | - |
| **130** | *Rickettsia japonica* YH | - | - | - |
| **131** | *Rickettsia massiliae* str. AZT80 | - | - | - |
| **132** | *Rickettsia montanensis str. OSU 85-930* | - | - | - |
| **133** | *Rickettsia parkeri* str. Portsmouth | - | - | - |
| **134** | *Rickettsia peacockii str. Rustic* | - | - | - |
| **135** | *Rickettsia philipii* str. 364D | - | - | - |
| **136** | *Rickettsia prowazekii* str. BuV67-CWPP | - | - | - |
| **137** | *Rickettsia rhipicephali* str. 3-7-female6-CWPP | - | - | - |
| **138** | *Rickettsia rickettsii* str. Arizona | - | - | - |
| **139** | *Rickettsia slovaca* 13-B | - | - | - |
| **140** | *Rickettsia typhi* str. Wilmington | - | - | - |
| **141** | *Wolbachia endosymbiont* of Culex quinquefasciatus Pel | - | - | - |
| **142** | *Wolbachia endosymbiont* of Drosophila melanogaster | - | - | - |
| **143** | *Wolbachia endosymbiont* of Onchocerca ochengi | - | - | - |
| **144** | *Wolbachia endosymbiont* strain TRS of Brugia malayi | - | - | - |
| **145** | *Wolbachia sp.* wRi | - | - | - |

There was a statistically significant difference ( * >+√8s_x_ and ** >+8s_x_)

***Supplementary Table S5. Genes carrying eight or more (AG)_3_-Di-SSRs in Vampire Pathogens (VP) of Mycoplasma, Rhizobiales and Rickettsiales***

| ***Mycoplasma* VP** | | |
| --- | --- | --- |
| **Locus** | **Product** | **Repeats** |
| WEN_03235 | ABC transporter | 9 |
| MSU_0708 | adenylosuccinate lyase | 8 |
| WEN_01175 | adenylosuccinate lyase | 8 |
| MHLP_00120 | DNA gyrase subunit A | 9 |
| MHLP_00950 | DNA helicase II | 10 |
| WEN_02870 | DNA ligase | 9 |
| MSU_0407 | DNA polymerase III PolC | 8 |
| MHLP_01025 | DNA polymerase III polC-type | 14 |
| Msui03470 | DNA polymerase III polC-type | 8 |
| WEN_02795 | DNA polymerase III polC-type | 14 |
| MHLP_04005 | DNA primase | 8 |
| WEN_03385 | DNA topoisomerase I | 8 |
| Msui06550 | DNA-directed RNA polymerase subunit beta | 12 |
| MHLP_03425 | DNA-directed RNA polymerase subunit beta' | 14 |
| WEN_01215 | DNA-directed RNA polymerase subunit beta' | 26 |
| MSU_0700 | DNA-directed RNA polymerase, beta' subunit | 11 |
| HF1_01030 | efflux ABC transporter permease | 8 |
| HF1_06800 | hypothetical protein | 8 |
| HF1_11030 | hypothetical protein | 8 |
| MHF_0752 | hypothetical protein | 8 |
| MHF_1196 | hypothetical protein | 8 |
| MHLP_00685 | hypothetical protein | 8 |
| MHLP_00690 | hypothetical protein | 8 |
| MHLP_00995 | hypothetical protein | 13 |
| WEN_01190 | hypothetical protein | 8 |
| WEN_02820 | hypothetical protein | 16 |
| MHLP_00580 | isoleucyl-tRNA ligase | 10 |
| WEN_00840 | isoleucyl-tRNA synthetase | 11 |
| MHLP_04260 | multifunctional aminopeptidase A | 8 |
| WEN_01500 | phenylalanyl-tRNA synthetase subunit beta | 8 |
| WEN_02665 | preprotein translocase subunit SecA | 11 |
| WEN_03395 | seryl-tRNA synthetase | 8 |
| MSU_0280 | type I site-specific deoxyribonuclease, HsdR family protein | 8 |
| WEN_02475 | type I site-specific deoxyribonuclease, HsdR family protein | 9 |
| MHLP_03150 | valyl-tRNA ligase | 9 |
| MHF_0136 | valyl-tRNA synthetase | 8 |
| WEN_00520 | valyl-tRNA synthetase | 9 |
|  |  |  |
| ***Rhizobiales* VP** | | |
| **Locus** | **Product** | **Repeats** |
| BQ00850 | cyclic beta 1-2 glucan synthetase | 8 |
| RM11_0081 | cyclic beta 1-2 glucan synthetase | 8 |
| Bgr_07860 | filamentous hemagglutinin | 8 |
| Bgr_15210 | filamentous hemagglutinin | 9 |
| Bgr_15780 | filamentous hemagglutinin | 8 |
| Btr_0394 | filamentous hemagglutinin | 8 |
| Btr_1297 | filamentous hemagglutinin | 8 |
| BAnh1_05790 | hypothetical protein | 9 |
| BH13120 | hypothetical protein | 14 |
| BH13430 | hypothetical protein | 8 |
| RM11_0959 | surface protein | 9 |
| BAnh1_02810 | surface protein/Bartonella adhesin | 18 |
|  |  |  |
|  | ***Rickettsiales* VP** |  |
| **Locus** | **Product** | **Repeats** |
| ACIS_00611 | hypothetical protein | 9 |
| ACIS_00764 | hypothetical protein | 10 |
| AM638 | hypothetical protein | 8 |
| AM712 | hypothetical protein | 8 |
| AMF_530 | hypothetical protein | 8 |
| AMF_395 | major surface protein 1a (MSP1A) | 9 |
| ACIS_00825 | NAD-glutamate dehydrogenase | 9 |

**Supplementary Table S6. The counts of SSRs in 145 genomes**

| **#** | **Name of Strains** | **Length of Repetitive Unit(bp)** | | | | | | |
| --- | --- | --- | --- | --- | --- | --- | --- | --- |
|  |  | **4** | **5** | **6** | **7** | **8** | **9** | **10** |
| ***Mycoplasma*** | | | | | | | | |
| **1** | *Mycoplasma agalactiae* | 33 | 1 | 2 | 0 | 0 | 0 | 0 |
| **2** | *Mycoplasma agalactiae* PG2 | 25 | 2 | 1 | 0 | 0 | 0 | 0 |
| **3** | *Mycoplasma arthritidis* 158L3-1 | 36 | 3 | 1 | 0 | 0 | 0 | 0 |
| **4** | *Mycoplasma bovis* HB0801 | 42 | 2 | 3 | 0 | 0 | 0 | 0 |
| **5** | *Mycoplasma bovis* Hubei-1 | 41 | 2 | 3 | 0 | 0 | 0 | 0 |
| **6** | *Mycoplasma bovis* PG45 | 42 | 3 | 2 | 0 | 0 | 0 | 0 |
| **7** | *Mycoplasma capricolum subsp. capricolum* ATCC 27343 | 50 | 6 | 5 | 1 | 0 | 0 | 0 |
| **8** | *Mycoplasma conjunctivae* HRC/581 | 25 | 1 | 1 | 0 | 0 | 2 | 0 |
| **9** | *Mycoplasma crocodyli* MP145 | 31 | 5 | 0 | 2 | 0 | 1 | 0 |
| **10** | *Mycoplasma fermentans* JER | 42 | 0 | 1 | 0 | 0 | 0 | 0 |
| **11** | *Mycoplasma fermentans* M64 | 60 | 0 | 1 | 0 | 0 | 0 | 0 |
| **12** | *Mycoplasma gallisepticum* NC06_2006.080-5-2P | 40 | 1 | 10 | 0 | 0 | 0 | 0 |
| **13** | *Mycoplasma gallisepticum* NC95_13295-2-2P | 40 | 2 | 12 | 0 | 0 | 0 | 0 |
| **14** | *Mycoplasma gallisepticum* NY01_2001.047-5-1P | 39 | 2 | 9 | 0 | 0 | 0 | 0 |
| **15** | *Mycoplasma gallisepticum* str. F | 44 | 1 | 7 | 0 | 0 | 0 | 0 |
| **16** | *Mycoplasma gallisepticum* str. R(high) | 42 | 2 | 14 | 0 | 0 | 0 | 0 |
| **17** | *Mycoplasma gallisepticum* str. R(low) | 42 | 2 | 13 | 0 | 0 | 0 | 0 |
| **18** | *Mycoplasma gallisepticum* VA94_7994-1-7P | 40 | 2 | 11 | 0 | 0 | 0 | 0 |
| **19** | *Mycoplasma gallisepticum* WI01_2001.043-13-2P | 39 | 1 | 9 | 0 | 0 | 0 | 0 |
| **20** | *Mycoplasma genitalium* G37 | 16 | 1 | 4 | 0 | 0 | 1 | 0 |
| **21** | *Mycoplasma genitalium* M2288 | 17 | 1 | 4 | 0 | 0 | 1 | 0 |
| **22** | *Mycoplasma genitalium* M2321 | 17 | 1 | 4 | 0 | 0 | 1 | 0 |
| **23** | *Mycoplasma genitalium* M6282 | 17 | 1 | 4 | 0 | 0 | 1 | 0 |
| **24** | *Mycoplasma genitalium* M6320 | 15 | 1 | 4 | 0 | 0 | 1 | 0 |
| **25** | *Mycoplasma haemocanis* str. Illinois | 6 | 0 | 22 | 5 | 1 | 2 | 2 |
| **26** | *Mycoplasma haemofelis* Ohio2 | 6 | 1 | 25 | 13 | 6 | 4 | 1 |
| **27** | *Mycoplasma haemofelis* str. Langford 1 | 6 | 1 | 31 | 17 | 9 | 11 | 2 |
| **28** | *Mycoplasma hominis* ATCC 23114 | 31 | 3 | 0 | 0 | 0 | 1 | 0 |
| **29** | *Mycoplasma hyopneumoniae* 168 | 28 | 7 | 10 | 2 | 1 | 1 | 0 |
| **30** | *Mycoplasma hyopneumoniae* 232 | 26 | 8 | 4 | 3 | 0 | 0 | 0 |
| **31** | *Mycoplasma hyopneumoniae* 7448 | 29 | 4 | 8 | 2 | 2 | 4 | 0 |
| **32** | *Mycoplasma hyopneumoniae* J | 28 | 10 | 6 | 3 | 1 | 1 | 0 |
| **33** | *Mycoplasma hyorhinis* GDL-1 | 43 | 2 | 4 | 1 | 0 | 0 | 0 |
| **34** | *Mycoplasma hyorhinis* HUB-1 | 40 | 2 | 5 | 1 | 0 | 0 | 0 |
| **35** | *Mycoplasma hyorhinis* MCLD | 43 | 2 | 4 | 1 | 0 | 0 | 0 |
| **36** | *Mycoplasma leachii* 99/014/6 | 59 | 5 | 4 | 0 | 0 | 1 | 0 |
| **37** | *Mycoplasma leachii* PG50 | 55 | 5 | 5 | 0 | 0 | 2 | 0 |
| **38** | *Mycoplasma mobile* 163K | 26 | 4 | 4 | 0 | 0 | 0 | 0 |
| **39** | *Mycoplasma mycoides subsp. capri* LC str. 95010 | 61 | 8 | 2 | 0 | 0 | 1 | 0 |
| **40** | *Mycoplasma mycoides subsp. capri* str. GM12 transgenic clone tetM-lacZ | 66 | 10 | 8 | 0 | 0 | 1 | 0 |
| **41** | *Mycoplasma mycoides subsp. mycoides* SC str. Gladysdale MU clone SC5 | 67 | 7 | 5 | 0 | 1 | 3 | 0 |
| **42** | *Mycoplasma mycoides subsp. mycoides* SC str. PG1 | 67 | 7 | 5 | 0 | 1 | 3 | 0 |
| **43** | *Mycoplasma penetrans* HF-2 | 68 | 5 | 2 | 0 | 0 | 0 | 0 |
| **44** | *Mycoplasma pneumoniae* 309 | 10 | 1 | 2 | 0 | 0 | 0 | 0 |
| **45** | *Mycoplasma pneumoniae* FH | 10 | 1 | 2 | 0 | 0 | 0 | 0 |
| **46** | *Mycoplasma pneumoniae* M129 | 9 | 1 | 2 | 0 | 0 | 0 | 0 |
| **47** | *Mycoplasma pulmonis* UAB CTIP | 36 | 5 | 7 | 0 | 0 | 0 | 0 |
| **48** | *Mycoplasma putrefaciens* KS1 | 33 | 5 | 3 | 0 | 0 | 0 | 0 |
| **49** | *Mycoplasma suis* KI3806 | 33 | 1 | 3 | 0 | 0 | 0 | 0 |
| **50** | *Mycoplasma suis* str. Illinois | 34 | 1 | 1 | 0 | 0 | 1 | 0 |
| **51** | *Mycoplasma synoviae* 53 | 26 | 3 | 4 | 2 | 0 | 1 | 0 |
| **52** | *Mycoplasma wenyonii* str. Massachusetts | 44 | 1 | 2 | 0 | 0 | 0 | 0 |
| **53** | *Candidatus Mycoplasma haemolamae* str. Purdue | 29 | 1 | 9 | 0 | 0 | 1 | 0 |
| ***Rhizobiales*** | | | | | | | | |
| **54** | *Agrobacterium fabrum* str. C58 | 37 | 4 | 5 | 0 | 0 | 0 | 0 |
| **55** | *Agrobacterium radiobacter* K84 | 52 | 10 | 7 | 0 | 0 | 0 | 0 |
| **56** | *Agrobacterium sp.* H13-3 | 36 | 6 | 2 | 1 | 0 | 0 | 0 |
| **57** | *Agrobacterium vitis* S4 chromosome 1 | 63 | 5 | 8 | 0 | 0 | 1 | 1 |
| **58** | *Azorhizobium caulinodans* ORS 571 | 136 | 7 | 11 | 2 | 0 | 1 | 0 |
| **59** | *Bartonella australis* Aust/NH1 | 21 | 2 | 1 | 0 | 0 | 0 | 0 |
| **60** | *Bartonella bacilliformis* KC583 | 21 | 1 | 2 | 2 | 2 | 0 | 0 |
| **61** | *Bartonella clarridgeiae* 73 | 28 | 8 | 5 | 0 | 0 | 5 | 0 |
| **62** | *Bartonella grahamii* as4aup | 34 | 3 | 5 | 0 | 2 | 1 | 0 |
| **63** | *Bartonella henselae* str. Houston-1 | 30 | 6 | 5 | 1 | 0 | 0 | 0 |
| **64** | *Bartonella quintana* RM-11 | 27 | 2 | 6 | 0 | 0 | 0 | 0 |
| **65** | *Bartonella quintana* str. Toulouse | 32 | 3 | 5 | 1 | 0 | 1 | 0 |
| **66** | *Bartonella tribocorum* CIP 105476 | 46 | 4 | 4 | 3 | 0 | 1 | 2 |
| **67** | *Bartonella vinsonii subsp. berkhoffii* str. Winnie | 34 | 3 | 2 | 1 | 2 | 2 | 0 |
| **68** | *Beijerinckia indica subsp. indica* ATCC 9039 | 53 | 7 | 7 | 1 | 0 | 0 | 0 |
| **69** | *Bradyrhizobium japonicum* USDA 6 | 92 | 18 | 23 | 0 | 0 | 0 | 1 |
| **70** | *Bradyrhizobium sp.* BTAi1 | 121 | 21 | 19 | 5 | 0 | 2 | 0 |
| **71** | *Bradyrhizobium sp.* ORS 278 | 92 | 16 | 13 | 4 | 0 | 3 | 0 |
| **72** | *Bradyrhizobium sp.* S23321 | 70 | 12 | 16 | 0 | 0 | 1 | 0 |
| **73** | *Brucella abortus* A13334 chromosome 1 | 33 | 4 | 2 | 0 | 7 | 1 | 0 |
| **74** | *Brucella canis* ATCC 23365 chromosome I | 31 | 5 | 2 | 0 | 7 | 1 | 0 |
| **75** | *Brucella melitensis* ATCC 23457 chromosome I | 31 | 6 | 1 | 0 | 5 | 0 | 0 |
| **76** | *Brucella microti* CCM 4915 chromosome 1 | 32 | 6 | 3 | 0 | 9 | 0 | 0 |
| **77** | *Brucella ovis* ATCC 25840 chromosome I | 31 | 6 | 1 | 1 | 6 | 0 | 0 |
| **78** | *Brucella pinnipedialis* B2/94 chromosome 1 | 33 | 6 | 2 | 0 | 9 | 0 | 0 |
| **79** | *Brucella suis* ATCC 23445 chromosome I | 29 | 4 | 4 | 0 | 9 | 0 | 0 |
| **80** | *Chelativorans sp.* BNC1 | 59 | 8 | 6 | 0 | 0 | 0 | 0 |
| **81** | *Hyphomicrobium denitrificans* ATCC 51888 | 52 | 7 | 10 | 3 | 1 | 0 | 0 |
| **82** | *Hyphomicrobium sp.* MC1 | 52 | 9 | 12 | 0 | 1 | 1 | 0 |
| **83** | *Mesorhizobium australicum* WSM2073 | 113 | 19 | 18 | 14 | 4 | 3 | 0 |
| **84** | *Mesorhizobium ciceri biovar biserrulae* WSM1271 | 124 | 12 | 23 | 2 | 22 | 3 | 0 |
| **85** | *Mesorhizobium loti* MAFF303099 | 114 | 12 | 17 | 12 | 4 | 2 | 0 |
| **86** | *Mesorhizobium opportunistum* WSM2075 | 128 | 19 | 15 | 19 | 4 | 2 | 0 |
| **87** | *Methylobacterium chloromethanicum* CM4 | 153 | 22 | 30 | 1 | 0 | 1 | 0 |
| **88** | *Methylobacterium extorquens* AM1 | 156 | 38 | 24 | 3 | 0 | 0 | 0 |
| **89** | *Methylobacterium nodulans* ORS 2060 | 290 | 59 | 47 | 3 | 2 | 3 | 0 |
| **90** | *Methylobacterium populi* BJ001 | 181 | 38 | 20 | 2 | 0 | 2 | 1 |
| **91** | *Methylobacterium radiotolerans* JCM 2831 | 273 | 65 | 41 | 1 | 1 | 2 | 2 |
| **92** | *Methylobacterium sp.* 4-46 | 494 | 106 | 76 | 5 | 1 | 6 | 1 |
| **93** | *Methylocella silvestris* BL2 | 72 | 41 | 12 | 1 | 1 | 1 | 0 |
| **94** | *Methylocystis sp.* SC2 | 48 | 38 | 18 | 1 | 2 | 1 | 0 |
| **95** | *Nitrobacter hamburgensis* X14 | 66 | 14 | 14 | 2 | 1 | 4 | 0 |
| **96** | *Nitrobacter winogradskyi* Nb-255 | 52 | 20 | 14 | 0 | 0 | 1 | 1 |
| **97** | *Ochrobactrum anthropi* ATCC 49188 chromosome 1 | 39 | 7 | 6 | 0 | 0 | 0 | 0 |
| **98** | *Oligotropha carboxidovorans* OM4 | 40 | 7 | 6 | 0 | 0 | 1 | 0 |
| **99** | *Parvibaculum lavamentivorans* DS-1 | 64 | 12 | 10 | 0 | 0 | 0 | 0 |
| **100** | *Pelagibacterium halotolerans* B2 | 52 | 9 | 6 | 0 | 0 | 1 | 0 |
| **101** | *Rhizobium etli* CFN 42 | 70 | 5 | 10 | 0 | 0 | 4 | 0 |
| **102** | *Rhizobium leguminosarum bv. trifolii* WSM1325 | 84 | 9 | 15 | 0 | 1 | 2 | 0 |
| **103** | *Rhizobium tropici* CIAT 899 | 44 | 2 | 6 | 3 | 0 | 1 | 1 |
| **104** | *Rhodomicrobium vannielii* ATCC 17100 | 39 | 18 | 10 | 1 | 1 | 3 | 0 |
| **105** | *Rhodopseudomonas palustris* BisA53 | 107 | 37 | 37 | 5 | 0 | 1 | 0 |
| **106** | *Sinorhizobium fredii* HH103 | 69 | 26 | 10 | 1 | 0 | 2 | 0 |
| **107** | *Sinorhizobium medicae* WSM419 | 55 | 13 | 11 | 2 | 0 | 1 | 0 |
| **108** | *Sinorhizobium meliloti* 1021 | 60 | 7 | 11 | 0 | 0 | 2 | 0 |
| **109** | *Starkeya novella* DSM 506 | 147 | 24 | 20 | 0 | 2 | 3 | 0 |
| **110** | *Xanthobacter autotrophicus* Py2 | 136 | 23 | 22 | 2 | 0 | 2 | 0 |
| ***Rickettsiales*** | | | | | | | | |
| **111** | *Anaplasma centrale* str. Israel | 8 | 2 | 0 | 0 | 0 | 0 | 0 |
| **112** | *Anaplasma marginale* str. Florida | 13 | 1 | 0 | 0 | 0 | 1 | 0 |
| **113** | *Anaplasma marginale* str. St. Maries | 12 | 1 | 0 | 0 | 0 | 0 | 0 |
| **114** | *Anaplasma phagocytophilum* HZ | 13 | 0 | 2 | 3 | 4 | 2 | 0 |
| **115** | *Candidatus Rickettsia amblyommii* str. GAT-30V | 41 | 1 | 2 | 0 | 0 | 0 | 0 |
| **116** | *Ehrlichia canis* str. Jake | 35 | 5 | 6 | 1 | 0 | 0 | 0 |
| **117** | *Ehrlichia chaffeensis* str. Arkansas | 21 | 6 | 1 | 1 | 0 | 0 | 0 |
| **118** | *Ehrlichia ruminantium* str. Gardel | 38 | 5 | 4 | 3 | 0 | 2 | 0 |
| **119** | *Neorickettsia risticii* str. Illinois | 13 | 0 | 0 | 0 | 0 | 0 | 0 |
| **120** | *Neorickettsia sennetsu* str. Miyayama | 14 | 1 | 0 | 0 | 0 | 0 | 0 |
| **121** | *Orientia tsutsugamushi* str. Boryong | 38 | 2 | 3 | 2 | 2 | 9 | 0 |
| **122** | *Rickettsia africae* ESF-5 | 39 | 5 | 2 | 0 | 0 | 0 | 0 |
| **123** | *Rickettsia akari* str. Hartford | 36 | 5 | 2 | 1 | 0 | 0 | 0 |
| **124** | *Rickettsia australis* str. Cutlack | 37 | 4 | 2 | 1 | 0 | 0 | 0 |
| **125** | *Rickettsia bellii* OSU 85-389 | 31 | 2 | 2 | 0 | 1 | 1 | 0 |
| **126** | *Rickettsia canadensis* str. CA410 | 33 | 4 | 1 | 1 | 2 | 1 | 0 |
| **127** | *Rickettsia conorii* str. Malish 7 | 39 | 4 | 1 | 0 | 0 | 0 | 0 |
| **128** | *Rickettsia felis* URRWXCal2 | 42 | 3 | 1 | 0 | 0 | 0 | 0 |
| **129** | *Rickettsia heilongjiangensis* 054 | 35 | 3 | 3 | 0 | 0 | 0 | 0 |
| **130** | *Rickettsia japonica* YH | 39 | 3 | 3 | 0 | 0 | 0 | 0 |
| **131** | *Rickettsia massiliae* str. AZT80 | 36 | 3 | 0 | 0 | 0 | 0 | 0 |
| **132** | *Rickettsia montanensis* str. OSU 85-930 | 37 | 5 | 0 | 0 | 0 | 1 | 0 |
| **133** | *Rickettsia parkeri* str. Portsmouth | 37 | 4 | 2 | 0 | 0 | 0 | 0 |
| **134** | *Rickettsia peacockii* str. Rustic | 39 | 5 | 1 | 0 | 0 | 0 | 0 |
| **135** | *Rickettsia philipii* str. 364D | 38 | 4 | 3 | 0 | 0 | 0 | 0 |
| **136** | *Rickettsia prowazekii* str. BuV67-CWPP | 33 | 5 | 1 | 0 | 0 | 0 | 0 |
| **137** | *Rickettsia rhipicephali* str. 3-7-female6-CWPP | 36 | 3 | 1 | 0 | 0 | 0 | 0 |
| **138** | *Rickettsia rickettsii* str. Arizona | 40 | 4 | 3 | 0 | 0 | 0 | 0 |
| **139** | *Rickettsia slovaca* 13-B | 40 | 5 | 2 | 1 | 0 | 0 | 0 |
| **140** | *Rickettsia typhi* str. Wilmington | 39 | 5 | 1 | 0 | 0 | 0 | 0 |
| **141** | *Wolbachia endosymbiont* of Culex quinquefasciatus Pel | 28 | 2 | 2 | 0 | 0 | 0 | 0 |
| **142** | *Wolbachia endosymbiont* of Drosophila melanogaster | 17 | 2 | 1 | 0 | 0 | 0 | 0 |
| **143** | *Wolbachia endosymbiont* of Onchocerca ochengi | 20 | 1 | 0 | 0 | 0 | 0 | 0 |
| **144** | *Wolbachia endosymbiont* strain TRS of Brugia malayi | 20 | 2 | 0 | 0 | 0 | 0 | 0 |
| **145** | *Wolbachia sp.* wRi | 15 | 2 | 2 | 0 | 0 | 0 | 0 |

**Supplementary Table S7. Significant analysis of the count of Mono-SSRs and Tri-SSRs between Vampire Pathogens (VP) and non Vampire Pathogens (N_VP) with Rank sum test (Mann-Whitney)**

| **Subtype of SSRs** | ***Mycoplasma*** | | | ***Rhizobiales*** | | | ***Rickettsiale*** | | |
| --- | --- | --- | --- | --- | --- | --- | --- | --- | --- |
|  | **VP** | **N-VP** | **P-value** | **VP** | **N-VP** | **P-value** | **VP** | **N-VP** | **P-value** |
| **Mono-SSRs** | | | | | | | | | |
| **(A/T)_n_-Mono-SSRs** | 1671.754 | 5604.318 | <0.001 | 3505.158 | 195.335 | <0.001 | 892.554 | 3407.224 | 0.005 |
| **(G/C)_n_-Mono-SSRs** | 254.569 | 22.940 | <0.001 | 170.855 | 104.443 | 0.064 | 304.382 | 29.724 | 0.005 |
| **Tri-SSRs** | | | | | | | | | |
| **(AAC)_n_-Tri-SSRs** | 55.494 | 102.516 | 0.003 | 86.029 | 11.549 | <0.001 | 53.225 | 40.650 | 0.133 |
| **(AAG)_n_-Tri-SSRs** | 197.863 | 81.511 | <0.001 | 106.387 | 43.425 | <0.001 | 40.751 | 97.850 | 0.005 |
| **(AAT)_n_-Tri-SSRs** | 35.372 | 289.125 | <0.001 | 92.596 | 2.442 | <0.001 | 9.980 | 371.970 | 0.005 |
| **(ACC)_n_-Tri-SSRs** | 12.689 | 25.733 | 0.002 | 43.000 | 39.297 | 0.364 | 48.427 | 17.226 | 0.005 |
| **(ACG)_n_-Tri-SSRs** | 2.643 | 0.988 | 0.010 | 7.261 | 126.756 | <0.001 | 11.643 | 3.370 | 0.005 |
| **(ACT)_n_-Tri-SSRs** | 26.425 | 21.896 | 0.193 | 3.132 | 1.582 | 0.001 | 17.534 | 46.692 | 0.005 |
| **(AGC)_n_-Tri-SSRs** | 15.218 | 27.937 | 0.005 | 72.434 | 96.129 | 0.005 | 104.787 | 37.720 | 0.005 |
| **(AGG)_n_-Tri-SSRs** | 36.957 | 4.338 | <0.001 | 17.939 | 23.415 | 0.039 | 29.939 | 11.041 | 0.005 |
| **(ATC)_n_-Tri-SSRs** | 46.527 | 85.035 | 0.007 | 85.372 | 57.753 | 0.005 | 29.108 | 54.424 | 0.005 |
| **(CCG)_n_-Tri-SSRs** | 0.000 | 0.941 | 1.000 | 7.211 | 416.425 | <0.001 | 32.434 | 10.931 | 0.005 |

The figures in VP and N_VP columns show the median of the counts of SSRs

**Supplementary Table S8. The basic information of all 145** chromosomes

| **#** | **Name of Strains** | **Genome Length**  **(Mbp)** | **GC%** | **Gene** | **Protein** | **Accession Number** | **Classification** |
| --- | --- | --- | --- | --- | --- | --- | --- |
| ***Mycoplasma*** | | | | | | | |
| **1** | *Mycoplasma agalactiae* | 1.01 | 29.6 | 866 | 814 | NC_013948.1 | [***Tenericutes/Mollicutes/Mycoplasmataceae/Mycoplasma***](http://www.ncbi.nlm.nih.gov/taxonomy/544448) |
| **2** | *Mycoplasma agalactiae* PG2 | 0.877438 | 29.7 | 768 | 666 | NC-009497.1 | [***Tenericutes/Mollicutes/Mycoplasmataceae/Mycoplasma***](http://www.ncbi.nlm.nih.gov/taxonomy/544448) |
| **3** | *Mycoplasma arthritidis* 158L3-1 | 0.820453 | 30.7 | 665 | 622 | NC_011025.1 | [***Tenericutes/Mollicutes/Mycoplasmataceae/Mycoplasma***](http://www.ncbi.nlm.nih.gov/taxonomy/544448) |
| **4** | *Mycoplasma bovis* HB0801 | 0.991702 | 29.3 | 841 | 776 | NC_018077.1 | ***Tenericutes/Mollicutes/Mycoplasmataceae/ Mycoplasma*** |
| **5** | *Mycoplasma bovis* Hubei-1 | 0.948121 | 29.3 | 812 | 742 | NC_015725.1 | ***Tenericutes/Mollicutes/Mycoplasmataceae/ Mycoplasma*** |
| **6** | *Mycoplasma bovis* PG45 | 1 | 29.3 | 868 | 814 | NC_014760.1 | ***Tenericutes/Mollicutes/Mycoplasmataceae/ Mycoplasma*** |
| **7** | *Mycoplasma capricolum subsp. capricolum* ATCC 27343 | 1.01 | 23.8 | 871 | 806 | NC_007633.1 | ***Tenericutes/Mollicutes/Mycoplasmataceae/Mycoplasma/Mycoplasma mycoides group/Mycoplasma capricolum*** |
| **8** | *Mycoplasma conjunctivae* HRC/581 | 0.846214 | 28.6 | 698 | 659 | NC_012806.1 | ***Tenericutes/Mollicutes/Mycoplasmataceae/ Mycoplasma*** |
| **9** | *Mycoplasma crocodyli* MP145 | 0.934379 | 27 | 792 | 747 | NC_014014.1 | [***Tenericutes/Mollicutes/Mycoplasmataceae/Mycoplasma***](http://www.ncbi.nlm.nih.gov/taxonomy/544448) |
| **10** | *Mycoplasma fermentans* JER | 0.977524 | 26.9 | 860 | 789 | NC_014552.1 | [***Tenericutes/Mollicutes/Mycoplasmataceae/Mycoplasma***](http://www.ncbi.nlm.nih.gov/taxonomy/544448) |
| **11** | *Mycoplasma fermentans* M64 | 1.12 | 26.9 | 1005 | 942 | NC_014921.1 | [***Tenericutes/Mollicutes/Mycoplasmataceae/Mycoplasma***](http://www.ncbi.nlm.nih.gov/taxonomy/544448) |
| **12** | *Mycoplasma gallisepticum* NC06_2006.080-5-2P | 0.938869 | 31.6 | 764 | 714 | NC_018411.1 | [***Tenericutes/Mollicutes/Mycoplasmataceae/Mycoplasma***](http://www.ncbi.nlm.nih.gov/taxonomy/544448) |
| **13** | *Mycoplasma gallisepticum* NC95_13295-2-2P | 0.953989 | 31.6 | 775 | 731 | NC_018411.1 | [***Tenericutes/Mollicutes/Mycoplasmataceae/Mycoplasma***](http://www.ncbi.nlm.nih.gov/taxonomy/544448) |
| **14** | *Mycoplasma gallisepticum* NY01_2001.047-5-1P | 0.965525 | 31.6 | 779 | 728 | NC_018409.1 | [***Tenericutes/Mollicutes/Mycoplasmataceae/Mycoplasma***](http://www.ncbi.nlm.nih.gov/taxonomy/544448) |
| **15** | *Mycoplasma gallisepticum* str. F | 0.977612 | 31.4 | 791 | 721 | NC_017503.1 | [***Tenericutes/Mollicutes/Mycoplasmataceae/Mycoplasma***](http://www.ncbi.nlm.nih.gov/taxonomy/544448) |
| **16** | *Mycoplasma gallisepticum* str. R(high) | 1.01 | 31.5 | 808 | 747 | [NC_017502.1](http://www.ncbi.nlm.nih.gov/nuccore/385325086) | [***Tenericutes/Mollicutes/Mycoplasmataceae/Mycoplasma***](http://www.ncbi.nlm.nih.gov/taxonomy/544448) |
| **17** | *Mycoplasma gallisepticum* str. R(low) | 1.01 | 31.5 | 811 | 753 | NC_004829.2 | [***Tenericutes/Mollicutes/Mycoplasmataceae/Mycoplasma***](http://www.ncbi.nlm.nih.gov/taxonomy/544448) |
| **18** | *Mycoplasma gallisepticum* VA94_7994-1-7P | 0.96411 | 31.6 | 784 | 740 | NC_018406.1 | [***Tenericutes/Mollicutes/Mycoplasmataceae/Mycoplasma***](http://www.ncbi.nlm.nih.gov/taxonomy/544448) |
| **19** | *Mycoplasma gallisepticum* WI01_2001.043-13-2P | 0.939844 | 31.6 | 768 | 721 | NC_018410.1 | [***Tenericutes/Mollicutes/Mycoplasmataceae/Mycoplasma***](http://www.ncbi.nlm.nih.gov/taxonomy/544448) |
| **20** | *Mycoplasma genitalium* G37 | 0.580076 | 31.7 | 558 | 509 | NC_000908.2 | [***Tenericutes/Mollicutes/Mycoplasmataceae/Mycoplasma***](http://www.ncbi.nlm.nih.gov/taxonomy/544448) |
| **21** | *Mycoplasma genitalium* M2288 | 0.579558 | 31.7 | 562 | 499 | NC_018498.1 | [***Tenericutes/Mollicutes/Mycoplasmataceae/Mycoplasma***](http://www.ncbi.nlm.nih.gov/taxonomy/544448) |
| **22** | *Mycoplasma genitalium* M2321 | 0.579977 | 31.7 | 562 | 492 | NC_018495.1 | [***Tenericutes/Mollicutes/Mycoplasmataceae/Mycoplasma***](http://www.ncbi.nlm.nih.gov/taxonomy/544448) |
| **23** | *Mycoplasma genitalium* M6282 | 0.579504 | 31.7 | 561 | 459 | NC_018496.1 | [***Tenericutes/Mollicutes/Mycoplasmataceae/Mycoplasma***](http://www.ncbi.nlm.nih.gov/taxonomy/544448) |
| **24** | *Mycoplasma genitalium* M6320 | 0.579796 | 31.7 | 565 | 495 | NC_018497.1 | [***Tenericutes/Mollicutes/Mycoplasmataceae/Mycoplasma***](http://www.ncbi.nlm.nih.gov/taxonomy/544448) |
| **25** | *Mycoplasma haemocanis* str. Illinois | 0.919992 | 35.3 | 1185 | 1132 | NC_016638.1 | [***Tenericutes/Mollicutes/Mycoplasmataceae/Mycoplasma***](http://www.ncbi.nlm.nih.gov/taxonomy/544448) |
| **26** | *Mycoplasma haemofelis* Ohio2 | 1.16 | 38.8 | 1583 | 1524 | NC_017520.1 | [***Tenericutes/Mollicutes/Mycoplasmataceae/Mycoplasma***](http://www.ncbi.nlm.nih.gov/taxonomy/544448) |
| **27** | *Mycoplasma haemofelis* str. Langford 1 | 1.15 | 38.9 | 1580 | 1545 | NC_014970.1 | [***Tenericutes/Mollicutes/Mycoplasmataceae/Mycoplasma***](http://www.ncbi.nlm.nih.gov/taxonomy/544448) |
| **28** | *Mycoplasma hominis* ATCC 23114 | 0.665445 | 27.1 | 590 | 541 | NC_013511.1 | [***Tenericutes/Mollicutes/Mycoplasmataceae/Mycoplasma***](http://www.ncbi.nlm.nih.gov/taxonomy/544448) |
| **29** | *Mycoplasma hyopneumoniae* 168 | 0.925576 | 28.5 | 732 | 685 | NC_017509.1 | [***Tenericutes/Mollicutes/Mycoplasmataceae/Mycoplasma***](http://www.ncbi.nlm.nih.gov/taxonomy/544448) |
| **30** | *Mycoplasma hyopneumoniae* 232 | 0.892758 | 28.6 | 710 | 657 | NC_006360.1 | [***Tenericutes/Mollicutes/Mycoplasmataceae/Mycoplasma***](http://www.ncbi.nlm.nih.gov/taxonomy/544448) |
| **31** | *Mycoplasma hyopneumoniae* 7448 | 0.920079 | 28.5 | 731 | 681 | NC_007332.1 | [***Tenericutes/Mollicutes/Mycoplasmataceae/Mycoplasma***](http://www.ncbi.nlm.nih.gov/taxonomy/544448) |
| **32** | *Mycoplasma hyopneumoniae* J | 0.897405 | 28.5 | 716 | 665 | NC_007295.1 | [***Tenericutes/Mollicutes/Mycoplasmataceae/Mycoplasma***](http://www.ncbi.nlm.nih.gov/taxonomy/544448) |
| **33** | *Mycoplasma hyorhinis* GDL-1 | 0.83748 | 25.9 | 775 | 698 | NC_016829.1 | [***Tenericutes/Mollicutes/Mycoplasmataceae/Mycoplasma***](http://www.ncbi.nlm.nih.gov/taxonomy/544448) |
| **34** | *Mycoplasma hyorhinis* HUB-1 | 0.839615 | 25.9 | 782 | 699 | NC_014448.1 | [***Tenericutes/Mollicutes/Mycoplasmataceae/Mycoplasma***](http://www.ncbi.nlm.nih.gov/taxonomy/544448) |
| **35** | *Mycoplasma hyorhinis* MCLD | 0.829709 | 25.9 | 770 | 678 | NC_017519.1 | ***Tenericutes/Mollicutes/Mycoplasmataceae/Mycoplasma*** |
| **36** | *Mycoplasma leachii* 99/014/6 | 1.02 | 23.7 | 916 | 858 | NC_017521.1 | ***Tenericutes/Mollicutes/Mycoplasmataceae/Mycoplasma*** |
| **37** | *Mycoplasma leachii* PG50 | 1.01 | 23.8 | 904 | 841 | NC_014751.1 | ***Tenericutes/Mollicutes/Mycoplasmataceae/Mycoplasma*** |
| **38** | *Mycoplasma mobile* 163K | 0.777079 | 25 | 688 | 654 | NC_006908.1 | [***Tenericutes/Mollicutes/Mycoplasmataceae/Mycoplasma***](http://www.ncbi.nlm.nih.gov/taxonomy/544448) |
| **39** | *Mycoplasma mycoides subsp. capri* LC str. 95010 | 1.15 | 23.8 | 980 | 927 | NC_015431.1 | ***Tenericutes/Mollicutes/Mycoplasmataceae/Mycoplasma/Mycoplasma mycoides group/ Mycoplasma mycoides/Mycoplasma mycoides subsp. Capri*** |
| **40** | *Mycoplasma mycoides subsp. capri* str. GM12 transgenic clone tetM-lacZ | 1.09 | 23.9 | 907 | 850 | NZ_CP001621.1 | ***Tenericutes/Mollicutes/Mycoplasmataceae/Mycoplasma/Mycoplasma mycoides group/ Mycoplasma mycoides/Mycoplasma mycoides subsp. Capri*** |
| **41** | *Mycoplasma mycoides subsp. mycoides* SC str. Gladysdale MU clone SC5 | 1.19 | 24 | 1093 | 984 | NC_021025.1 | ***Tenericutes/Mollicutes/Mycoplasmataceae/Mycoplasma/Mycoplasma mycoides group/ Mycoplasma mycoides/Mycoplasma mycoides subsp. Capri*** |
| **42** | *Mycoplasma mycoides subsp. mycoides* SC str. PG1 | 1.21 | 24 | 1053 | 1017 | NC_005364.2 | ***Tenericutes/Mollicutes/Mycoplasmataceae/Mycoplasma/Mycoplasma mycoides group/ Mycoplasma mycoides/Mycoplasma mycoides subsp. Capri*** |
| **43** | *Mycoplasma penetrans* HF-2 | 1.36 | 25.7 | 1065 | 1024 | NC_004432.1 | [***Tenericutes/Mollicutes/Mycoplasmataceae/Mycoplasma***](http://www.ncbi.nlm.nih.gov/taxonomy/544448) |
| **44** | *Mycoplasma pneumoniae* 309 | 0.817176 | 40 | 774 | 710 | NC_016807.1 | [***Tenericutes/Mollicutes/Mycoplasmataceae/Mycoplasma***](http://www.ncbi.nlm.nih.gov/taxonomy/544448) |
| **45** | *Mycoplasma pneumoniae* FH | 0.811088 | 40 | 768 | 684 | NC_017504.1 | [***Tenericutes/Mollicutes/Mycoplasmataceae/Mycoplasma***](http://www.ncbi.nlm.nih.gov/taxonomy/544448) |
| **46** | *Mycoplasma pneumoniae* M129 | 0.816394 | 40 | 1061 | 691 | NC_000912.1 | [***Tenericutes/Mollicutes/Mycoplasmataceae/Mycoplasma***](http://www.ncbi.nlm.nih.gov/taxonomy/544448) |
| **47** | *Mycoplasma pulmonis* UAB CTIP | 0.963879 | 26.6 | 786 | 739 | NC_002771.1 | [***Tenericutes/Mollicutes/Mycoplasmataceae/Mycoplasma***](http://www.ncbi.nlm.nih.gov/taxonomy/544448) |
| **48** | *Mycoplasma putrefaciens* KS1 | 0.832603 | 26.9 | 713 | 647 | NC_015946.1 | [***Tenericutes/Mollicutes/Mycoplasmataceae/Mycoplasma***](http://www.ncbi.nlm.nih.gov/taxonomy/544448) |
| **49** | *Mycoplasma suis* KI3806 | 0.70927 | 31.1 | 814 | 772 | NC_015153.1 | [***Tenericutes/Mollicutes/Mycoplasmataceae/Mycoplasma***](http://www.ncbi.nlm.nih.gov/taxonomy/544448) |
| **50** | *Mycoplasma suis* str. Illinois | 0.742431 | 31.1 | 864 | 808 | NC_015155.1 | [***Tenericutes/Mollicutes/Mycoplasmataceae/Mycoplasma***](http://www.ncbi.nlm.nih.gov/taxonomy/544448) |
| **51** | *Mycoplasma synoviae* 53 | 0.799476 | 28.5 | 720 | 645 | NC_007294.1 | [***Tenericutes/Mollicutes/Mycoplasmataceae/Mycoplasma***](http://www.ncbi.nlm.nih.gov/taxonomy/544448) |
| **52** | *Mycoplasma wenyonii* str. Massachusetts | 0.650228 | 33.9 | 713 | 678 | NC_018149.1 | [***Tenericutes/Mollicutes/Mycoplasmataceae/Mycoplasma***](http://www.ncbi.nlm.nih.gov/taxonomy/544448) |
| **53** | *Candidatus Mycoplasma haemolamae* str. Purdue | 0.756845 | 39.3 | 961 | 925 | NC_018219.1 | [***Tenericutes/Mollicutes/Mycoplasmataceae/Mycoplasma***](http://www.ncbi.nlm.nih.gov/taxonomy/544448) |
| ***Rhizobiales*** | | | | | | | |
| **54** | *Agrobacterium fabrum* str. C58 | 2.84 | 59.4 | 2819 | 2765 | NC_003062.2 | ***Proteobacteria/Alphaproteobacteria/ Rhizobiales/Rhizobiaceae/Rhizobium/Agrobacterium group/Agrobacterium/Agrobacterium tumefaciens complex*** |
| **55** | *Agrobacterium radiobacter* K84 | 4.01 | 60.3 | 3889 | 3801 | NC_011985.1 | ***Proteobacteria/Alphaproteobacteria/ Rhizobiales/Rhizobiaceae/Rhizobium/Agrobacterium group/Agrobacterium/Agrobacterium tumefaciens complex*** |
| **56** | *Agrobacterium sp.* H13-3 | 2.82 | 58.8 | 2751 | 2688 | NC_015183.1 | ***Proteobacteria/Alphaproteobacteria/ Rhizobiales/Rhizobiaceae/Rhizobium/Agrobacterium group/Agrobacteriu*** |
| **57** | *Agrobacterium vitis* S4 chromosome 1 | 3.73 | 57.7 | 3479 | 3406 | NC_011989.1 | ***Proteobacteria/Alphaproteobacteria/ Rhizobiales/Rhizobiaceae/Rhizobium/Agrobacterium group/Agrobacteriu*** |
| **58** | *Azorhizobium caulinodans* ORS 571 | 5.37 | 67.3 | 4834 | 4740 | NC_009937.1 | ***Proteobacteria/Alphaproteobacteria/Rhizobiales/Xanthobacteraceae/ Azorhizobium*** |
| **59** | *Bartonella australis* Aust/NH1 | 1.6 | 41.8 | 1400 | 1321 | NC_020300.1 | ***Proteobacteria/Alphaproteobacteria/Rhizobiales/Bartonellaceae/ Bartonella*** |
| **60** | *Bartonella bacilliformis* KC583 | 1.45 | 38.2 | 1268 | 1188 | NC_008783.1 | ***Proteobacteria/Alphaproteobacteria/Rhizobiales/Bartonellaceae/Bartonella/Bartonella bacilliformis*** |
| **61** | *Bartonella clarridgeiae* 73 | 1.52 | 35.7 | 1277 | 1188 | NC_014932.1 | ***Proteobacteria/Alphaproteobacteria/Rhizobiales/Bartonellaceae/ Bartonella*** |
| **62** | *Bartonella grahamii* as4aup | 2.34 | 38.1 | 1977 | 1839 | NC_012846.1 | ***Proteobacteria/Alphaproteobacteria/Rhizobiales/Bartonellaceae/ Bartonella*** |
| **63** | *Bartonella henselae* str. Houston-1 | 1.93 | 38.2 | 1683 | 1532 | NC_005956.1 | ***Proteobacteria/Alphaproteobacteria/Rhizobiales/Bartonellaceae/ Bartonella*** |
| **64** | *Bartonella quintana* RM-11 | 1.59 | 38.8 | 1356 | 1206 | NC_018533.1 | ***Proteobacteria/Alphaproteobacteria/Rhizobiales/Bartonellaceae/ Bartonella*** |
| **65** | *Bartonella quintana* str. Toulouse | 1.58 | 38.8 | 1355 | 1224 | NC_005955.1 | ***Proteobacteria/Alphaproteobacteria/Rhizobiales/Bartonellaceae/Bartonella/Bartonella quintana*** |
| **66** | *Bartonella tribocorum* CIP 105476 | 2.62 | 38.9 | 2360 | 2208 | NC_010161.1 | ***Proteobacteria/Alphaproteobacteria/Rhizobiales/Bartonellaceae/ Bartonella*** |
| **67** | *Bartonella vinsonii subsp. berkhoffii* str. Winnie | 1.8 | 38.8 | 1644 | 1486 | NC_020301.1 | ***Proteobacteria/Alphaproteobacteria/Rhizobiales/Bartonellaceae/Bartonella/ Bartonella vinsonii/Bartonella vinsonii subsp. berkhoffii*** |
| **68** | *Beijerinckia indica subsp. indica* ATCC 9039 | 4.17 | 57.1 | 3740 | 3614 | NC_010581.1 | ***Proteobacteria/Alphaproteobacteria/Rhizobiales/Beijerinckiaceae/ Beijerinckia*** |
| **69** | *Bradyrhizobium japonicum* USDA 6 | 9.21 | 63.7 | 8483 | 8233 | NC_017249.1 | ***Proteobacteria/Alphaproteobacteria/Rhizobiales/Bradyrhizobiaceae/Bradyrhizobium/Bradyrhizobium japonicum*** |
| **70** | *Bradyrhizobium sp.* BTAi1 | 8.26 | 64.9 | 7379 | 7251 | NC_009485.1 | ***Proteobacteria/Alphaproteobacteria/Rhizobiales/Bradyrhizobiaceae/ Bradyrhizobium*** |
| **71** | *Bradyrhizobium sp.* ORS 278 | 7.46 | 65.5 | 6641 | 6532 | NC_009445.1 | ***Proteobacteria/Alphaproteobacteria/Rhizobiales/Bradyrhizobiaceae/ Bradyrhizobium*** |
| **72** | *Bradyrhizobium sp.* S23321 | 7.23 | 64.3 | 6779 | 6713 | NC_017082.1 | ***Proteobacteria/Alphaproteobacteria/Rhizobiales/Bradyrhizobiaceae/ Bradyrhizobium*** |
| **73** | *Brucella abortus* A13334 chromosome 1 | 2.12 | 57.2 | 2086 | 2026 | NC_016795.1 | ***Proteobacteria/Alphaproteobacteria/Rhizobiales/Brucellaceae/Brucella/Brucella abortus*** |
| **74** | *Brucella canis* ATCC 23365 chromosome I | 2.11 | 57.2 | 2022 | 1940 | NC_010103.1 | ***Proteobacteria/Alphaproteobacteria/Rhizobiales/Brucellaceae/Brucella/ Brucella canis*** |
| **75** | *Brucella melitensis* ATCC 23457 chromosome I | 2.13 | 57.2 | 2059 | 1977 | NC_012441.1 | ***Proteobacteria/Alphaproteobacteria/Rhizobiales/Brucellaceae/Brucella/ Brucella melitensis/Brucella melitensis bv. 2*** |
| **76** | *Brucella microti* CCM 4915 chromosome 1 | 2.12 | 57.2 | 2024 | 1950 | NC_013119.1 | ***Proteobacteria/Alphaproteobacteria/Rhizobiales/Brucellaceae/Brucella*** |
| **77** | *Brucella ovis* ATCC 25840 chromosome I | 2.11 | 57.2 | 2068 | 1949 | NC_009505.1 | ***Proteobacteria/Alphaproteobacteria/Rhizobiales/Brucellaceae/Brucella/Brucella ovis*** |
| **78** | *Brucella pinnipedialis* B2/94 chromosome 1 | 2.14 | 57.2 | 2081 | 2005 | NC_015857.1 | ***Proteobacteria/Alphaproteobacteria/Rhizobiales/Brucellaceae/Brucella/ Brucella pinnipedialis*** |
| **79** | *Brucella suis* ATCC 23445 chromosome I | 1.92 | 57.1 | 1848 | 1773 | NC_010169.1 | ***Proteobacteria/Alphaproteobacteria/Rhizobiales/Brucellaceae/Brucella/Brucella suis/Brucella suis bv. 2*** |
| **80** | *Chelativorans sp.* BNC1 | 4.41 | 61.1 | 4167 | 4075 | NC_008254.1 | ***Proteobacteria/Alphaproteobacteria/Rhizobiales/Phyllobacteriaceae/Chelativorans*** |
| **81** | *Hyphomicrobium denitrificans* ATCC 51888 | 3.64 | 60.8 | 3524 | 3463 | NC_014313.1 | ***Proteobacteria/Alphaproteobacteria/Rhizobiales/Hyphomicrobiaceae/Hyphomicrobium/Hyphomicrobium denitrificans*** |
| **82** | *Hyphomicrobium sp.* MC1 | 4.76 | 59.2 | 4594 | 4512 | NC_015717.1 | ***Proteobacteria/Alphaproteobacteria/Rhizobiales/Hyphomicrobiaceae/ Hyphomicrobium*** |
| **83** | *Mesorhizobium australicum* WSM2073 | 6.2 | 62.8 | 5910 | 5762 | NC_019973.1 | ***Proteobacteria/Alphaproteobacteria/Rhizobiales/Phyllobacteriaceae/Mesorhizobium*** |
| **84** | *Mesorhizobium ciceri biovar biserrulae* WSM1271 | 6.26 | 62.7 | 6100 | 5870 | NC_014923.1 | ***Proteobacteria/Alphaproteobacteria/Rhizobiales/Phyllobacteriaceae/Mesorhizobium/Mesorhizobium ciceri/Mesorhizobium ciceri biovar biserrulae*** |
| **85** | *Mesorhizobium loti* MAFF303099 | 7.04 | 62.7 | 6759 | 6590 | NC_002678.2 | ***Proteobacteria/Alphaproteobacteria/Rhizobiales/Phyllobacteriaceae/Mesorhizobium*** |
| **86** | *Mesorhizobium opportunistum* WSM2075 | 6.88 | 62.9 | 6583 | 6438 | NC_015675.1 | ***Proteobacteria/Alphaproteobacteria/Rhizobiales/Phyllobacteriaceae/Mesorhizobium*** |
| **87** | *Methylobacterium chloromethanicum* CM4 | 5.51 | 68.7 | 5071 | 4949 | NC_012808.1 | ***Proteobacteria/Alphaproteobacteria/Rhizobiales/Methylobacteriaceae/Methylobacterium/Methylobacterium extorquens group/Methylobacterium extorquens*** |
| **88** | *Methylobacterium extorquens* AM1 | 5.78 | 68.2 | 5252 | 5087 | NC_011757.1 | ***Proteobacteria/Alphaproteobacteria/Rhizobiales/Methylobacteriaceae/Methylobacterium/Methylobacterium extorquens group/Methylobacterium extorquens*** |
| **89** | *Methylobacterium nodulans* ORS 2060 | 7.77 | 68.9 | 7266 | 7024 | NC_011894.1 | ***Proteobacteria/Alphaproteobacteria/Rhizobiales/Methylobacteriaceae/Methylobacterium*** |
| **90** | *Methylobacterium populi* BJ001 | 5.8 | 69.4 | 5360 | 5253 | NC_010725.1 | ***Proteobacteria/Alphaproteobacteria/Rhizobiales/Methylobacteriaceae/Methylobacterium*** |
| **91** | *Methylobacterium radiotolerans* JCM 2831 | 6.08 | 71.5 | 5691 | 5584 | NC_010505.1 | ***Proteobacteria/Alphaproteobacteria/Rhizobiales/Methylobacteriaceae/Methylobacterium*** |
| **92** | *Methylobacterium sp.* 4-46 | 7.66 | 71.6 | 6744 | 6542 | NC_010511.1 | ***Proteobacteria/Alphaproteobacteria/Rhizobiales/Methylobacteriaceae/Methylobacterium*** |
| **93** | *Methylocella silvestris* BL2 | 4.31 | 63.1 | 3984 | 3866 | NC_011666.1 | ***Proteobacteria/Alphaproteobacteria/Rhizobiales/Beijerinckiaceae/Methylocella*** |
| **94** | *Methylocystis sp.* SC2 | 3.77 | 63.4 | 3654 | 3575 | NC_018485.1 | ***Proteobacteria/Alphaproteobacteria/Rhizobiales/Methylocystaceae/Methylocystis*** |
| **95** | *Nitrobacter hamburgensis* X14 | 4.41 | 61.7 | 4147 | 3956 | NC_007964.1 | ***Proteobacteria/Alphaproteobacteria/Rhizobiales/Bradyrhizobiaceae/Nitrobacter*** |
| **96** | *Nitrobacter winogradskyi* Nb-255 | 3.4 | 62 | 3270 | 3165 | NC_007406.1 | ***Proteobacteria/Alphaproteobacteria/Rhizobiales/Bradyrhizobiaceae/Nitrobacter*** |
| **97** | *Ochrobactrum anthropi* ATCC 49188 chromosome 1 | 2.89 | 56.1 | 2858 | 2796 | NC_009667.1 | ***Proteobacteria/Alphaproteobacteria/Rhizobiales/Brucellaceae/Ochrobactrum*** |
| **98** | *Oligotropha carboxidovorans* OM4 | 3.54 | 62.5 | 3367 | 3299 | NC_017538.1 | ***Proteobacteria/Alphaproteobacteria/Rhizobiales/Bradyrhizobiaceae/Oligotropha/Oligotropha carboxidovorans*** |
| **99** | *Parvibaculum lavamentivorans* DS-1 | 3.91 | 62.3 | 3715 | 3648 | NC_009719.1 | ***Proteobacteria/Alphaproteobacteria/Rhizobiales/Rhodobiaceae/Parvibaculum*** |
| **100** | *Pelagibacterium halotolerans* B2 | 3.94 | 61.4 | 3854 | 3772 | NC_016078.1 | ***Proteobacteria/Alphaproteobacteria/Rhizobiales/Hyphomicrobiaceae/Pelagibacterium*** |
| **101** | *Rhizobium etli* CFN 42 | 4.38 | 61.3 | 4219 | 4076 | NC_007761.1 | ***Proteobacteria/Alphaproteobacteria/Rhizobiales/Rhizobiaceae/Rhizobium/Agrobacterium group/Rhizobium*** |
| **102** | *Rhizobium leguminosarum bv. trifolii* WSM1325 | 4.77 | 61.1 | 4609 | 4505 | NC_012850.1 | ***Proteobacteria/Alphaproteobacteria/Rhizobiales/Rhizobiaceae/Rhizobium/Agrobacterium group/Rhizobium/Rhizobium leguminosaru*** |
| **103** | *Rhizobium tropici* CIAT 899 | 3.84 | 59.9 | 3721 | 3644 | NC_020059.1 | ***Proteobacteria/Alphaproteobacteria/Rhizobiales/Rhizobiaceae/Rhizobium/Agrobacterium group/Rhizobium*** |
| **104** | *Rhodomicrobium vannielii* ATCC 17100 | 4.01 | 62.2 | 3702 | 3607 | NC_014664.1 | ***Proteobacteria/Alphaproteobacteria/Rhizobiales/Hyphomicrobiaceae/Rhodomicrobium*** |
| **105** | *Rhodopseudomonas palustris* BisA53 | 5.51 | 64.4 | 4921 | 4838 | NC_008435.1 | ***Proteobacteria/Alphaproteobacteria/Rhizobiales/Bradyrhizobiaceae/Rhodopseudomonas*** |
| **106** | *Sinorhizobium fredii* HH103 | 4.31 | 62.6 | 4050 | 3951 | NC_016812.1 | ***Proteobacteria/Alphaproteobacteria/Rhizobiales/Rhizobiaceae/Sinorhizobium/Ensifer group/Sinorhizobium/Sinorhizobium fredii group*** |
| **107** | *Sinorhizobium medicae* WSM419 | 3.78 | 61.5 | 3634 | 3529 | NC_009636.1 | ***Proteobacteria/Alphaproteobacteria/Rhizobiales/Rhizobiaceae/Sinorhizobium/Ensifer group/Sinorhizobium*** |
| **108** | *Sinorhizobium meliloti* 1021 | 3.65 | 62.7 | 3429 | 3359 | NC_003047.1 | ***Proteobacteria/Alphaproteobacteria/Rhizobiales/Rhizobiaceae/Sinorhizobium/Ensifer group/ Sinorhizobium*** |
| **109** | *Starkeya novella* DSM 506 | 4.77 | 67.9 | 4485 | 4412 | NC_014217.1 | ***Proteobacteria/Alphaproteobacteria/Rhizobiales/Xanthobacteraceae/Starkeya*** |
| **110** | *Xanthobacter autotrophicus* Py2 | 5.31 | 67.5 | 4855 | 4761 | NC_009720.1 | ***Proteobacteria/Alphaproteobacteria/Rhizobiales/Xanthobacteraceae/Xanthobacter*** |
| ***Rickettsiales*** | | | | | | | |
| **111** | *Anaplasma centrale* str. Israel | 1.21 | 50 | 1012 | 953 | NC_013532.1 | ***Proteobacteria/Alphaproteobacteria/Rickettsiales/Anaplasmataceae/Anaplasma*** |
| **112** | *Anaplasma marginale* str. Florida | 1.2 | 49.8 | 1013 | 961 | NC_012026.1 | ***Proteobacteria/Alphaproteobacteria/Rickettsiales/Anaplasmataceae/Anaplasma*** |
| **113** | *Anaplasma marginale* str. St. Maries | 1.2 | 49.8 | 1015 | 959 | NC_004842.2 | ***Proteobacteria/Alphaproteobacteria/Rickettsiales/Anaplasmataceae/Anaplasma*** |
| **114** | *Anaplasma phagocytophilum* HZ | 1.47 | 41.6 | 1145 | 1080 | NC_007797.1 | ***Proteobacteria/Alphaproteobacteria/Rickettsiales/Anaplasmataceae/Anaplasma*** |
| **115** | *Candidatus Rickettsia amblyommii* str. GAT-30V | 1.41 | 32.4 | 1476 | 1158 | NC_017028.1 | ***Proteobacteria/Alphaproteobacteria/Rickettsiales/Rickettsiaceae/Rickettsieae/Rickettsia/spotted fever group*** |
| **116** | *Ehrlichia canis* str. Jake | 1.32 | 29 | 972 | 929 | NC_007354.1 | ***Proteobacteria/Alphaproteobacteria/Rickettsiales/Anaplasmataceae/Ehrlichia/canis group*** |
| **117** | *Ehrlichia chaffeensis* str. Arkansas | 1.18 | 1.18 | 936 | 884 | NC_007799.1 | ***Proteobacteria/Alphaproteobacteria/Rickettsiales/Anaplasmataceae/Ehrlichia/canis group*** |
| **118** | *Ehrlichia ruminantium* str. Gardel | 1.5 | 27.5 | 962 | 913 | NC_006831.1 | ***Proteobacteria/Alphaproteobacteria/Rickettsiales/Anaplasmataceae/Ehrlichia/canis group*** |
| **119** | *Neorickettsia risticii* str. Illinois | 0.879977 | 41.3 | 810 | 761 | NC_013009.1 | ***Proteobacteria/Alphaproteobacteria/Rickettsiales/Anaplasmataceae/Neorickettsia*** |
| **120** | *Neorickettsia sennetsu* str. Miyayama | 0.859006 | 41.1 | 794 | 754 | NC_007798.1 | ***Proteobacteria/Alphaproteobacteria/Rickettsiales/Anaplasmataceae/Neorickettsia*** |
| **121** | *Orientia tsutsugamushi* str. Boryong | 2.13 | 30.5 | 2008 | 1593 | NC_009488.1 | ***Proteobacteria/Alphaproteobacteria/Rickettsiales/Rickettsiaceae/Rickettsieae/ Orientia*** |
| **122** | *Rickettsia africae* ESF-5 | 1.28 | 32.4 | 1400 | 1229 | NC_012633.1 | ***Proteobacteria/Alphaproteobacteria/Rickettsiales/Rickettsiaceae/Rickettsieae/Rickettsia/spotted fever group*** |
| **123** | *Rickettsia akari* str. Hartford | 1.23 | 32.3 | 1228 | 992 | NC_009881.1 | ***Proteobacteria/Alphaproteobacteria/Rickettsiales/Rickettsiaceae/Rickettsieae/Rickettsia/spotted fever group*** |
| **124** | *Rickettsia australis* str. Cutlack | 1.3 | 32.3 | 1341 | 1138 | NC_017058.1 | ***Proteobacteria/Alphaproteobacteria/Rickettsiales/Rickettsiaceae/Rickettsieae/Rickettsia/spotted fever group*** |
| **125** | *Rickettsia bellii* OSU 85-389 | 1.53 | 31.6 | 1516 | 1391 | NC_009883.1 | ***Proteobacteria/Alphaproteobacteria/Rickettsiales/Rickettsiaceae/Rickettsieae/Rickettsia/belli group/Rickettsia bellii*** |
| **126** | *Rickettsia canadensis* str. CA410 | 1.15 | 31 | 987 | 876 | NC_016929.1 | ***Proteobacteria/Alphaproteobacteria/Rickettsiales/Rickettsiaceae/Rickettsieae/Rickettsia/belli group/Rickettsia canadensis*** |
| **127** | *Rickettsia conorii* str. Malish 7 | 1.27 | 32.4 | 1371 | 1234 | NC_003103.1 | ***Proteobacteria/Alphaproteobacteria/Rickettsiales/Rickettsiaceae/Rickettsieae/Rickettsia/spotted fever group*** |
| **128** | *Rickettsia felis* URRWXCal2 | 1.49 | 32.5 | 1482 | 1309 | NC_007109.1 | ***Proteobacteria/Alphaproteobacteria/Rickettsiales/Rickettsiaceae/Rickettsieae/Rickettsia/spotted fever group*** |
| **129** | *Rickettsia heilongjiangensis* 054 | 1.28 | 32.3 | 1389 | 1174 | NC_015866.1 | ***Proteobacteria/Alphaproteobacteria/Rickettsiales/Rickettsiaceae/Rickettsieae/Rickettsia/spotted fever group*** |
| **130** | *Rickettsia japonica* YH | 1.28 | 32.4 | 1399 | 1184 | NC_016050.1 | ***Proteobacteria/Alphaproteobacteria/Rickettsiales/Rickettsiaceae/Rickettsieae/Rickettsia/spotted fever group*** |
| **131** | *Rickettsia massiliae* str. AZT80 | 1.26 | 32.6 | 1345 | 1087 | NC_016931.1 | ***Proteobacteria/Alphaproteobacteria/ Rickettsiales/Rickettsiaceae/ Rickettsieae/ Rickettsia/spotted fever group/Rickettsia massiliae*** |
| **132** | *Rickettsia montanensis* str. OSU 85-930 | 1.28 | 32.6 | 1380 | 1173 | NC_017043.1 | ***Proteobacteria/Alphaproteobacteria/Rickettsiales/Rickettsiaceae/Rickettsieae/Rickettsia/spotted fever group*** |
| **133** | *Rickettsia parkeri* str. Portsmouth | 1.3 | 32.4 | 1396 | 1243 | NC_017044.1 | ***Proteobacteria/Alphaproteobacteria/Rickettsiales/Rickettsiaceae/Rickettsieae/Rickettsia/spotted fever group*** |
| **134** | *Rickettsia peacockii* str. Rustic | 1.29 | 32.6 | 964 | 927 | NC_012730.1 | ***Proteobacteria/Alphaproteobacteria/Rickettsiales/Rickettsiaceae/Rickettsieae/Rickettsia/spotted fever group*** |
| **135** | *Rickettsia philipii* str. 364D | 1.29 | 32.5 | 1359 | 1235 | NC_016930.1 | ***Proteobacteria/Alphaproteobacteria/Rickettsiales/Rickettsiaceae/Rickettsieae/Rickettsia/spotted fever group*** |
| **136** | *Rickettsia prowazekii* str. BuV67-CWPP | 1.11 | 29 | 897 | 855 | NC_017056.1 | ***Proteobacteria/Alphaproteobacteria/Rickettsiales/Rickettsiaceae/Rickettsieae/Rickettsia/typhus group/ Rickettsia prowazekii*** |
| **137** | *Rickettsia rhipicephali* str. 3-7-female6-CWPP | 1.29 | 32.4 | 1412 | 1134 | NC_017042.1 | ***Proteobacteria/Alphaproteobacteria/Rickettsiales/Rickettsiaceae/Rickettsieae/Rickettsia/spotted fever group*** |
| **138** | *Rickettsia rickettsii* str. Arizona | 1.27 | 32.4 | 1353 | 1225 | NC_016909.1 | ***Proteobacteria/Alphaproteobacteria/Rickettsiales/Rickettsiaceae/Rickettsieae/Rickettsia/spotted fever group*** |
| **139** | *Rickettsia slovaca* 13-B | 1.28 | 32.5 | 1410 | 1271 | NC_016639.1 | ***Proteobacteria/Alphaproteobacteria/Rickettsiales/Rickettsiaceae/Rickettsieae/Rickettsia/spotted fever group*** |
| **140** | *Rickettsia typhi* str. Wilmington | 1.11 | 28.9 | 865 | 818 | NC_006142.1 | ***Proteobacteria/Alphaproteobacteria/Rickettsiales/Rickettsiaceae/Rickettsieae/Rickettsia/typhus group*** |
| **141** | *Wolbachia endosymbiont* of Culex quinquefasciatus Pel | 1.48 | 34.2 | 1380 | 1296 | NC_010981.1 | ***Proteobacteria/Alphaproteobacteria/Rickettsiales/Anaplasmataceae/Wolbachieae/ Wolbachia/Wolbachia endosymbiont of Culex quinquefasciatus*** |
| **142** | *Wolbachia endosymbiont* of Drosophila melanogaster | 1.27 | 35.2 | 1198 | 1074 | NC_002978.6 | ***Proteobacteria/Alphaproteobacteria/Rickettsiales/Anaplasmataceae/Wolbachieae/ Wolbachia*** |
| **143** | *Wolbachia endosymbiont* of Onchocerca ochengi | 0.95799 | 32.1 | 730 | 649 | NC_018267.1 | ***Proteobacteria/Alphaproteobacteria/Rickettsiales/Anaplasmataceae/Wolbachieae/ Wolbachia*** |
| **144** | *Wolbachia endosymbiont* strain TRS of Brugia malayi | 1.08 | 34.2 | 993 | 805 | NC_006833.1 | ***Proteobacteria/Alphaproteobacteria/Rickettsiales/Anaplasmataceae/Wolbachieae/ Wolbachia*** |
| **145** | *Wolbachia sp.* wRi | 1.45 | 35.2 | 1355 | 1258 | NC_012416.1 | ***Proteobacteria/Alphaproteobacteria/Rickettsiales/Anaplasmataceae/Wolbachieae/ Wolbachia*** |
